# Endogenous Retroviruses Activity as a Molecular Signature of Neurodevelopmental Disorders

**DOI:** 10.3390/ijms20236050

**Published:** 2019-11-30

**Authors:** Emanuela Balestrieri, Claudia Matteucci, Chiara Cipriani, Sandro Grelli, Laura Ricceri, Gemma Calamandrei, Paola Sinibaldi Vallebona

**Affiliations:** 1 Department of Experimental Medicine, University of Rome Tor Vergata, 00133 Rome, Italy; matteucci@med.uniroma2.it (C.M.); chiaracipriani88@gmail.com (C.C.); grelli@med.uniroma2.it (S.G.); sinibaldi-vallebona@med.uniroma2.it (P.S.V.); 2 Centre for Behavioural Sciences and Mental Health, Istituto Superiore di Sanità, 00161 Rome, Italy; laura.ricceri@iss.it (L.R.); gemma.calamandrei@iss.it (G.C.); 3 Institute of Translational Pharmacology, National Research Council, 00133 Rome, Italy

**Keywords:** endogenous retroviruses (ERVs), autism, neurodevelopmental disorders, maternal immune activation, ASD animal model

## Abstract

Human endogenous retroviruses (HERVs) are genetic elements resulting from relics of ancestral infection of germline cells, now recognized as cofactors in the etiology of several complex diseases. Here we present a review of findings supporting the role of the abnormal HERVs activity in neurodevelopmental disorders. The derailment of brain development underlies numerous neuropsychiatric conditions, likely starting during prenatal life and carrying on during subsequent maturation of the brain. Autism spectrum disorders, attention deficit hyperactivity disorders, and schizophrenia are neurodevelopmental disorders that arise clinically during early childhood or adolescence, currently attributed to the interplay among genetic vulnerability, environmental risk factors, and maternal immune activation. The role of HERVs in human embryogenesis, their intrinsic responsiveness to external stimuli, and the interaction with the immune system support the involvement of HERVs in the derailed neurodevelopmental process. Although definitive proofs that HERVs are involved in neurobehavioral alterations are still lacking, both preclinical models and human studies indicate that the abnormal expression of ERVs could represent a neurodevelopmental disorders-associated biological trait in affected individuals and their parents.

## 1. Human Endogenous Retroviruses

### 1.1. The Origin of the Human Endogenous Retroviruses

The completion of the human genome project [1] has allowed the subsequent important discovery that more than half was composed of mobile genomic elements, named transposable elements [2,3]. Among them, DNA transposons move by a cut-and-paste mechanism [4], while retrotransposons mobilize by a copy-and-paste mechanism via an RNA intermediate [5]. The retrotransposons comprise long terminal repeats (LTR)-elements, namely human endogenous retroviruses (HERVs) [6], and non-LTR elements, which include long and short interspersed nuclear elements (LINEs and SINEs, respectively) [7]. LINEs, moving autonomously, are widely spread within the genome [1] and also supply reverse transcriptase (RT) for the retro transposition of other endogenous retroelements [8].

HERVs are derived from their exogenous retroviral counterpart by a process of germline infection and proliferation within the host genome [9,10], and their integration as proviruses led to the fixation and the vertical transmission, following Mendelian laws [11]. Endogenous retrovirus sequences are highly represented within the mammalian genomes, as they account for 5% to 10% of the genetic material [1,12] in different species [13]. The differences between species are due to subsequent independent invasions of the mammalian genome by different viral progenitors, which occurred during the evolution of the mammals themselves, so each species is unique in its content of endogenous retrovirus sequences [14,15]. The phylogenetic analysis demonstrated that the human genome holds approximately one hundred thousand different HERVs loci [16], resulting from the proliferation of a few initial germline invasions by exogenous retroviruses [9,17,18,19]. The mechanisms by which HERVs proliferated into the human genome, increasing their copy number, are still yet to be fully clarified and it has been proposed that the retrotransposition within germline cells, recombination events [20], as well as new reinfections [9], could account for the spread of HERVs sequences. As a consequence, extensive interindividual variation due to new insertions (known for the family HERV-K) [21], dimorphism for various families (HERV-K, HERV-W, and HERV-H) [20], and unfixed copies [22,23], has been demonstrated. As a consequence, during their co-evolution with humans, the genomic structure of HERVs, resembling the exogenous counterpart since it is composed of *gag, pro*, *pol* and *env* genes and two flanked LTRs [24], has been substantially altered. Mutations, deletions, and sequence rearrangements, accumulated in most HERVs, resulted in the loss of coding and infectious capacity [25]. HERV-K (HML-2), the most recently endogenized HERVs group, is instead present as full-length copies, likely to be insertionally polymorphic between individuals [26].

### 1.2. Physiological Functions of HERVs

Given their abundance in the human genome, HERVs represent an important source of genomic variability, also providing potential coding and regulatory elements for the acquisition of new cellular functions [27,28,29,30]. Indeed, due to the long co-evolution with humans, some HERVs have been coopted for physiological functions [28,29] while their reactivation in response to external stimuli has been associated with human pathological conditions [31,32,33].

A significant amount of evidence has been obtained regarding the general expression of HERVs in normal tissues [34,35], and several mechanisms account for their contribution to the host genome structure and function and to the physiological effects on the human transcriptome. An age-related transcriptional activity of HERV-H, HERV-K, and HERV-W has been observed in peripheral blood mononuclear cells (PBMCs) from a large cohort of healthy human subjects aged between 1 and 80 years, reinforcing the hypothesis of a physiological correlation between HERVs activity and the different stages of life in humans [36]. 

Among the proposed mechanisms by which HERVs could contribute to the human physiology, it is recognized that various sequences, concentrated in the LTRs, are involved in the regulation of the expression of neighboring genes since they serve as promoters [37], enhancers [38], and polyadenylation signals [39], as regulators of chromatin folding [40] and as binding sites for transcriptional factors [41]. Most HERVs reside in the genome as solo-LTRs, resulting from homologous recombination between the LTRs of a full-length HERV [42] and, interestingly, recombination events among different HERVs may determine genomic instability [43].

LTRs can also act as alternative tissue-specific promoters to drive the expression of host genes [44,45,46] HERVs sequences are also engaged by the host for the regulation of gene expression in embryo development [47]. Indeed, non-coding RNA (ncRNAs) expressed by the HERV-H group and the recruitment of specific cellular transcriptional factors on HERV-H LTRs seems to be involved in the conservation of stem cell identity [41,48]. Of note, the HERV-H loci seem to be more preserved in a full-length state than other HERVs families, suggesting that the full-length elements rather than solo-LTRs are useful to the host and that the internal regions of HERV-H may be involved in the process of exaptation [49]. Similarly, an ancestral *env* gene dubbed HEMO [human endogenous MER34 (medium reiteration-frequency-family-34) ORF] has been found highly expressed in embryos, already in the early stages of development, and in all subsequent differentiation periods as well as in the placenta and in the blood of pregnant women [50]. A pivotal role in the placental syncytiotrophoblast development and homeostasis and in the maternal immunetolerance to the paternal antigens on the fetus is played by the syncytin-1 and 2, Env proteins of HERV-W and HERV-FRD, respectively [51,52,53]. Syncytin-1 promotes cell fusion, similar to the Env protein of an exogenous viral counterpart, while syncytin-2 is involved in maternal tolerance, with a mechanism not yet clarified [54]. The lack of syncytins expression, caused by hypermethylation, was reported to be associated with various placental abnormalities [55].

### 1.3. HERVs Responsiveness to Environmental Stimuli and their Deregulation in Human Diseases

In the dynamic regulation of HERV expression from embryonic to differentiated cells, these elements have been shown to be regulated by epigenetic mechanisms. In terminally differentiated somatic cells, HERV expression is silenced through DNA methylation and histone modifications; otherwise, their aberrant reactivation threatens genomic integrity, resulting in the development of diseases. Indeed, HERV sequences conserve some of their pathological properties, contributing to the development of several human complex diseases, such as cancer, inflammatory diseases, and neurological and psychiatric disorders [32,33,56,57,58]. However, so far, an unequivocal pathogenic cause-effect relationship has not been established. Although the mechanisms leading to HERV repression/activation are not fully elucidated, a variety of environmental stimuli of different natures, such as microorganisms, cytokines, hormones, vitamins, nutrients, and drugs, have been involved in HERV transactivation.

Of particular relevance is the role played by the interaction with microbes, including viruses, exogenous retroviruses, intestinal microbiota, and protozoan. Indeed, several viruses have been demonstrated to deregulate HERV activity, which in turn contributes to the development of viral diseases, including virus-associated tumors [59]. Among the herpesviruses, HSV-1 induces HERV-W *gag* and *env* transcripts in epithelial and astrocytic cancer cell lines [60] and the production of the HERV-W Gag and Env proteins in neuroblastoma cells [61]. The Epstein Barr virus (EBV) induces human endogenous retrovirus, such as W/Multiple Sclerosis-associated retroviruses (HERV-W/MSRV), syncytin-1 transcript and protein in astrocytes, B cells and monocytes [62], and HERV-K18 Env protein, in primary B cells [63]. A genome-wide activation of LTR enhancers and promoters has been reported during the EBV-induced transformation of B cells, suggesting an impact on host gene regulatory networks [64]. Moreover, the involvement of HERVs in Kaposi sarcoma has been suggested since KSHV *de novo* infection or latent viral proteins are able to induce the expression of the oncogenic Np9 protein of HERV-K [65]. Human herpesvirus 6 (HHV-6) infection has long been suspected of playing a role in the pathogenesis of multiple sclerosis (MS) and neuroinflammation, and recently it has been demonstrated that HHV-6A induces the expression of HERV-W/MSRV Env protein that in turn may play a role in the inflammatory process [66]. Moreover, high expression of HERV-K and HERV-H transcripts was found in colon samples from inflammatory bowel diseases (IBD) patients affected by herpesvirus infections when compared with those not infected [67].

HERVs have been suggested as cofactors also in Hepatitis B virus-associated hepatocellular carcinoma since the oncogenic protein HBx can to upregulate the expression of HERV-W *env* transcripts through NF-κB in transformed liver cells [68]. HERVs activation could also be involved in HTLV-1 associated diseases as adult T cell leukemia and tropical spastic paraparesis since the oncogenic protein Tax activates HERV-LTRs [69]. Many recent studies have shown that infection with the human immunodeficiency virus-1 (HIV-1) is able to transactivate HERV-K (HML2) expression both in vivo and in vitro, suggesting this group as a cofactor in acquired immune deficiency syndrome (AIDS) and AIDS-associated cancers [70,71]. In particular, the HIV-1 Tat protein has been shown to induce HERV-K *gag*, *rec*, and *Np9* transcripts in acute leukemia T cell line and in primary lymphocytes [72] and HERV-W/MSRV *env* transcripts and proteins in human astrocyte and blood cells [73].

Influenza A virus can induce transactivation of the *env* gene in the HERV-W locus ERVWE1 by increasing the transcription factor glial cells missing 1 and reducing the repressive histone mark H3K9me3 [74]. The HERVs transactivation has also been observed in neuronal cells infected by *Toxoplasma gondii* [75], and it has been hypothesized that the imbalances of the intestinal microbiota is able to modulate the transcription of retroelements [76]. Moreover, the in vitro exposure of PBMCs to lipopolysaccharide (LPS) or interferon-γ (IFN-γ) modulated HERV/MaLR transcriptome [77]. Interestingly, the presence of interferon-stimulated response elements (ISREs) in the promoter region of HERVs suggests that HERV-K expression could be regulated by inflammatory cytokines in amyotrophic lateral sclerosis (ALS), and this mechanism could be extended to other diseases, in which the inflammation plays a role in the pathogenesis [78]. Furthermore, cytokines, such as tumor necrosis factor-alpha (TNF-α), interleukin-1alpha (IL-1α), interleukin-1beta (IL-1β), and IFN-γ, can modulate HERV-R *env* gene expression in vascular endothelial cells [79].

It is also known that the HERV expression is modulated by endogenous hormones, both in pathological and physiological conditions. Indeed, cyclic modification of the expression of HERVs in reproductive-age females suggests regulation of HERV-K expression levels by sex hormones progesterone and estradiol [80]. The same hormones increase HERV-K *env* transcripts in breast cancer cell lines [81] and pituitary adenomas cells [82], while in patients affected by systemic lupus erythematosus steroid treatment decreased HERV clone 4-1 *gag* mRNA expression [83].

Stimuli affecting HERVs expression come from mitogens, such as phytohemagglutinin (PHA) and phorbol-12-myristate-13-acetate (PMA), in T cells [84]. PMA can also induce the release of virus-like particles associated with the increased transcriptional expression of several HERVs in macrophages and monocytoid cells [85]. Nutrients intake and vitamins can also affect HERVs activity through epigenetic mechanisms [86,87], and the in vitro starvation induces HERV-K transcriptional expression and the release of viral particles in melanoma cells [88,89,90].

Drugs can also influence the expression of HERVs. The anticancer agent etoposide has been found to upregulate several HERVs transcripts and proteins in chemo resistant colon carcinomas cells [91], while inhibitors of the histone deacetylase (HDACi), such as valproic acid (VPA), have been demonstrated to increase the transcriptional expression of HERVs in brain cell lines [92]. Moreover, upregulation of HERV-W and ERV9 transcription was detected in *post mortem* brain from patients with schizophrenia previously treated with VPA [92]. Vorinostat treatment modulates the HERVs transcriptional activity in primary CD4^+^T cells [93], while decitabine and benzene-derived metabolite hydroquinone induce epigenetic switches that activate HERVs in monocytic leukemia cell lines and hematopoietic stem cells [94].

Among environmental insults, ultraviolet irradiations (UV) induce the transcriptional activation of HERV-K *pol* gene as well as the expression of Env protein in melanoma cells [95]. Likewise, treatment with UVB or 5-aza-deoxycytidine induces increased HERV-E mRNA expression in CD4^+^ T cells from patients with systemic lupus erythematosus (SLE) [96].

Hence, HERVs have been found particularly responsive to environmental stimuli that can determine a dysregulation of HERVs transcription and/or HERV-encoded protein expression that could influence the phenotype of complex diseases. For instance, high HERV transcripts and proteins have been found in tumor tissues, and antibodies against HERVs have been detected in sera from patients [32,97,98]. HERVs involvement in the tumorigenesis process could occur through different mechanisms, such as insertional mutagenesis, activation of downstream oncogenes, expression of the HERV-K oncogenic proteins Np9 and Rec, alteration of cellular checkpoints, fusogenic and immunosuppressive activity [32]. For this reason, their targeting has been proposed for the treatment of aggressive cancers [99].

HERVs are suggested to also be involved in autoimmunity and inflammatory diseases, such as multiple sclerosis (MS) and rheumatoid arthritis (RA). HERV-W Env protein, identified in brain lesions from MS patients, is able to induce a proinflammatory response in human macrophages cells through toll-like receptor (TLR) 4 pathway activation [100]. On this basis, a humanized monoclonal antibody targeting the HERV-W/MSRV Env protein has been developed as an innovative therapeutic approach for MS [101].

In RA patients, a significantly elevated IgG antibody response to HERV-K10 Gag matrix peptide was observed [102] while, in the cartilage of patients with osteoarthritis, HERV-W activity was confirmed by high expression levels of syncytin, dsRNA, virus budding, and the presence of virus-like particles, suggesting the involvement of HERVs in the etiopathogenesis of both the diseases [103]. HERVs are also considered to be involved in type 1 diabetes, an association with HERV K-18 polymorphisms [104], and a significant increase in HERV-W *env* mRNA in PBMCs and Env protein expression in sera and in pancreatic biopsies of patients have been observed [105]. These data are further supported by a preclinical study in transgenic mice expressing HERV-W Env protein, displaying hyperglycemia and decreased levels of insulin, and by in vitro experiments demonstrating that HERV-W Env protein directly inhibits insulin secretion in human Langerhans islets [105].

Moreover, growing evidence supports the involvement of HERVs in neurodevelopmental disorders, such as autism spectrum disorders (ASD), attention deficit hyperactivity disorder (ADHD), and schizophrenia (SCZ) (see Section 3).

## 2. Neurodevelopmental Disorders

### 2.1. Main Clinical and Genetic Features of Neurodevelopmental Disorders

The derailment of brain development underlies numerous neuropsychiatric conditions, and the pathological events likely start during prenatal life and carry on during subsequent maturation of the brain [106,107].

ASD, ADHD, and SCZ are neurodevelopmental disorders (NDDs) that arise clinically mostly during early childhood or adolescence, and share clinical features, vulnerability genes, and environmental risk factors. ASD is a complex neurodevelopmental disorder characterized by symptoms, including persistent challenges in social interaction, speech, and nonverbal communication, and restricted/repetitive behaviors, whose severity is different in each individual. The first diagnosis usually occurs in childhood, although many signs are already evident in the first six months of life, suggesting that ASD pathogenesis begins early during the development [108]. ADHD is one of the most common childhood mental disorders, also affecting many adults. Symptoms of ADHD comprise inattention, hyperactivity, and impulsivity [108]. SCZ is a chronic brain disorder, and when active, symptoms include delusions, hallucinations, trouble with thinking and concentration, and lack of motivation [108]. While disease onset typically occurs in late adolescence or early adulthood, several lines of evidence suggest that SCZ results from aberrations occurring in fetal development [109]. These neurodevelopmental disorders seem to have other clinical features in common [110,111]: the risk of ADHD is higher in ASD families [112], and ADHD is also the most common comorbidity in autistic patients [113], as well as in the past, ASD has been defined as an early manifestation of SCZ, since one-third of childhood-onset cases first received a diagnosis of ASD [110], and ASD was also associated with SCZ among siblings of probands with ASD [114].

Mounting evidence demonstrated that ASD, ADHD, and SCZ share partially overlapping sets of common genetic risk factors [115,116,117]. In particular, some key gene sets, implicated in the post-synaptic density, in central nervous system development, neural projections, and synaptic transmission, and in various other cellular processes, were shared by two or more disorders, including ASD, ADHD, SCZ, anxiety disorders, bipolar disease (BD) and major depressive disorder [118,119]. Moreover, copy number variation (CNV) of the DOCK8/KANK1 locus was found in SCZ, BD, ASD, and ADHD patients, suggesting that shared structural variants could contribute to explain the genetic basis for co-morbidity and co-occurrence of these disorders [120].

### 2.2. Drugs and Infections Exposure in Sensitive Time-Windows of the Pregnancy as Risk Factors for NDDs

Although there is a strong genetic component, several environmental factors act to increase the risk of NDDs by a neurotoxic mechanism [121,122,123]. Exogenous elements, ranging from environmental toxicants (methyl mercury, leads, arsenic, pesticides, polychlorinated biphenyls, and bisphenol A) to maternal intake of drugs (thalidomide, valproic acid, misoprostol, selective serotonin reuptake inhibitors) as well as maternal infections, can alter neurodevelopment. They act mostly between conception and birth, in specific well-delineated sensitive time-windows of increased vulnerability of the nervous system (see Heyer and Meredith, 2017 for a comprehensive review of environmental toxicants in NDDs) [122].

Among the intake of drugs during pregnancy, the anticonvulsant valproic acid (VPA) has been associated with an increased risk of somatic anomalies, ASD, and other developmental disabilities in the offspring [124,125]. VPA is widely used for the treatment of bipolar disorders, migraine, headaches, neuropathic pain, and especially epilepsy. Since seizures have been associated with the risk of miscarriage and low birth weight, women with epilepsy require an antiepileptic treatment for the entire length of the pregnancy [126].

VPA intake in pregnancy can induce teratogenic effects, including, besides neural tube defects, limb, cardiovascular, and craniofacial anomalies, and the association between the VPA exposure during the first trimester of pregnancy and reduced cognitive functions and high risk for ASD among the offspring has been reported [127].

To explain the therapeutic effects of VPA, as well as its neurotoxicity, multiple mechanisms are called upon: direct interference with GABAergic neurotransmission, interaction with neural remodeling and neurogenesis, modulation of folate metabolism, free radicals production, interference with cell proliferation/migration patterns and alterations of inflammatory and immunologic markers [127,128,129,130,131]. Animal studies demonstrated that VPA also acts on the regulation of gene expression via epigenetic mechanisms, since it is a non-selective inhibitor of histone deacetylase of class I and II (HDAC1 and HDAC2), resulting in the modulation of several genes and proteins implicated in neuronal excitation and inhibition and in brain and immune system development [128,132,133,134]. Moreover, Choi and co-authors showed the transgenerational non-genetic inheritance of the autism-like phenotype in mice prenatally exposed to VPA [135].

Infections occurring in pregnancy can also determine long-lasting changes in the brain and, therefore, could affect behavior in the offspring. Rubella infection in pregnancy has been associated with a high incidence of neurological abnormalities and with an increased prevalence of intellectual disability and autism [136], especially if the infection occurred in the second month of pregnancy [137]. Congenital rubella infection also increases later risk of SCZ; in this case, the effect is strongest whether infection happened during the first two gestational months [138] or not. Respiratory infections by influenza virus during the first trimester of pregnancy have also been shown to increase the incidence of SCZ in the offspring [139], while maternal genito–urinary tract infections (chlamydia urethritis, trichomoniasis, and candidiasis) were associated with significantly increased risk of ADHD [140], depending on the time-window of the pregnancy in which infection occurs [141]. Moreover, the hospitalization of the mother during pregnancy has been associated with an increased ASD risk in the offspring, both for viral infections in the first trimester and for bacterial infections in the second one [142], further highlighting that the real risk factor is the time-window of the infection. Other infectious agents have been associated with ASD risk in offspring, including cytomegalovirus [143], polyomaviruses [144], and influenza virus [145,146].

### 2.3. Inflammatory Mechanisms in NDDs

This consistent amount of data strongly supports a relationship between maternal infection and derailed neurodevelopment in the offspring, underlying the potential role of the maternal immune response to pathogens in determining NDDs.

In pregnancy, the inflammation induced by several insults could affect different stages of neurodevelopment in the fetu*s*, contributing to the appearance of altered phenotypes early in childhood as well as adult or aged progeny [147,148].

In animal models, this concept is strongly supported by the maternal immune activation (MIA) model, obtained by challenging pregnant rodents via direct infection (e.g., influenza virus, *Escherichia coli*) or with the viral mimic polyinosinic–polycytidylic acid [Poly(I:C)], a synthetic analog of double-stranded RNA (dsRNA). In the MIA model, prenatal exposure to these insults leads to significant immunological, neurodevelopmental, and behavioral changes in the offspring [149,150,151]. It is notable that the maternal immune response rather than the infection/insult itself was responsible for the aberrant phenotype in the offspring [152,153].

Growing evidence pointed out the role of inflammation in the pathogenesis of NDDs since the deregulation of the innate and adaptive immune responses can affect brain function and development, highlighting the close interconnection between the immune and nervous systems. Abnormal cytokines levels in sera and amniotic fluid were reported [154,155], and an association with a particular ASD sub-phenotype was proposed [156]. Maternal inflammatory cytokines, induced by MIA, could cross the placenta or cytokines, could be produced *in loco* in the placenta itself, with a consequent gene dysregulation in the fetus [155]. Moreover, MIA could promote the production of pro-inflammatory cytokines, not only in the mother but also in the developing brain and body of the fetus [157], leading to permanent dysregulation of the immune system in the offspring [158]. In line with this hypothesis, in autistic patients high levels of cytokines, such as interleukin (IL)-1β, IL-5, IL-6, IL-8, IL-12, IL-13, IL-17, IL-23, TNF-α and INF-γ were found in blood and in cerebrospinal fluid (see Masi et al., 2017 for a comprehensive review) [159] and our research group has recently demonstrated high expression levels of TNF-α, IFN-γ, and IL-10 in PBMCs of autistic children and their mothers [160]. Acute neuroinflammation during early fetal development may be relevant also for ADHD since in patients elevated concentrations of the pro-inflammatory cytokine TNF-β and reduced levels of anti-inflammatory cytokines IL-2, IL-4, and IFN-γ have been reported [161]. Additionally, inflammatory and immune system diseases of mothers in pregnancy have been associated with an increased risk of ADHD in their offspring [162]. Finally, increased pro-inflammatory cytokines in patients with established SCZ [163] as well as in drug-naïve patients with first-episode psychosis [164] and in individuals at high risk of psychosis [165] were observed, suggesting that the dysregulation of the innate immune system occurs early in the onset of the SCZ. It is noteworthy that during the acute psychotic relapses, high serum levels of IL-6 and TNF-α were detected, and the reduction of IL-6 levels after antipsychotic treatment was observed [166].

## 3. HERVs in Neurodevelopmental Disorders

### 3.1. Evidence from Human Studies on the Involvement of HERVs in Neurodevelopmental Disorders

Based on current findings, complex neurodevelopmental disorders, such as ASD, ADHD, and SCZ, seem to be the result of the interplay among genetic vulnerability, environmental risk factors, and maternal immune activation. All these factors act during prenatal life, but how they contribute to derailing neurological development has to be elucidated.

Growing evidence demonstrates an abnormal RNA expression and protein production of several HERVs in NDDs, in which HERVs could be involved spanning the bridge between genetic susceptibility and environmental risk factors. However, the significance of the altered transcriptional activity, as well as the potentially detrimental effect of HERV-encoded proteins, has yet to be fully clarified.

Studies on ADHD and ASD patients showed altered transcriptional activity of the *env* gene of several HERVs in the PBMCs of the patients in comparison to age- and sex-matched healthy control individuals [167,168,169]. In particular, the expression of the RNA of the HERV-H *env* gene was higher in PBMCs from autistic patients than in healthy controls, while the expression of HERV-W *env* was higher in control children [167,169]. Moreover, the in vitro exposure to phytohemagglutinin (PHA) and IL-2 induced the expression of HERV-H in PBMCs from autistic patients but not of healthy controls, suggesting an intrinsic potential to express HERV-H *env* gene of PBMCs from patients [167]. In addition, HERV-H expression has been found to be negatively correlated with the age of autistic patients and associated with more severe clinical signs, suggesting altered HERV-H transcriptional activity as a disease-dependent feature [167]. Recently, in a study including autistic patients and their parents, we describe that children and their mothers share abnormal RNA levels of HERV-H *env* gene, of HEMO and of the cytokines TNF-α, IFN-γ, and IL-10, suggesting a close mother–child association within families with autistic children [160]. It is worthy of notice that both in patients and mothers, the expression of HEMO correlated with TNF-α [160], supporting the hypothesis of an interplay between HERVs and the innate immune response. Considering the functions of HERV-H and the potential role of HEMO in human embryogenesis, their abnormal expression could contribute to the derailed neurodevelopmental process as cofactorsor at least the altered expression of HERVs and cytokines could be considered as a molecular signature of ASD that permits the discernment of affected children and their mothers from healthy controls.

In addition, in PBMCs from drug-naïve ADHD patients, abnormal expression of RNA of HERV-H *env* gene has been observed, supporting the view that the transcriptional activation of this specific retroviral element might represent a distinctive trait of the disorder [168]. As with observed autistic patients, in ADHD children, the expression of HERV-H was closely related to the symptomatology, being more expressed in children with severe symptoms and increased in response to in vitro stimulation with PHA and IL-2 [168]. Notably, ADHD patients in therapy with methylphenidate (MPH) displayed a fast reduction in HERV-H activity in parallel with an improvement in clinical symptoms, making candidate HERV-H a potential predictive marker of the response to MPH therapy [170,171].

Accumulating evidence highlights the association between HERVs and schizophrenia as there is an increase in HERV-W *gag* and *env* genes and ERV9 *pol* transcript and protein in blood samples of subjects with schizophrenia [172,173,174,175]. In addition, transcripts of HERV-K10 were found increased in *post-mortem* brain tissue from patients with schizophrenia and bipolar disorders [176] and in line, retroviral RNAs of HERV-W, ERV9, and HERV-FRD have been identified in the cerebral spinal fluid of schizophrenia patients [177]. HERV-W Gag proteins levels were found to be decreased in the anterior cingulate gyrus and the hippocampus [178], whereas they were detected, together with HERV-W Env proteins, in serum samples of individuals with schizophrenia and correlated with C-reactive protein levels [179]. The disparity between these reports may be due to different experimental methods or anti-psychotic treatments in SCZ patients. Interestingly, in patients with schizophrenia, the levels of HERV-W transcripts are related to the activity of immune components, such as proinflammatory cytokines, often dysregulated in the disease [180]. It is also known that epigenetic processes, including DNA methylation, are involved in schizophrenia [181], and significantly lower levels of HERV-K methylation in patients with first-episode schizophrenia in comparison with controls were reported. Otherwise, patients with multi-episode schizophrenia had HERV-K methylation levels similar to those in controls. The authors hypothesized that the findings in patients with multi-episode schizophrenia might be attributed to the effects of antipsychotic treatment since they observed a positive correlation between the dosage of antipsychotics and the levels of HERV-K methylation [182].

The potential involvement of HERVs in NDDs could be due to their intrinsic responsiveness to external stimuli, described above; environmental stimuli of different natures may be translated into the cell as gene expression regulation through several epigenetic mechanisms [183].

### 3.2. Mechanisms by Which HERVsCouldDerail Neurodevelopment

Since several HERV families play important roles in mammalian development and differentiation [50,184,185], their abnormal activity could have an impact on the different stages of embryogenesis. Embryonic development is driven through epigenetic regulation, leading to global remodeling, cell commitment, and tissue specification [186]. During this sensitive phase, any alteration could impact on neurodevelopment [187], and, therefore, epigenetic modifications could directly link molecular regulatory pathways to environmental factors and explain some aspects of complex disorders, such as ASD, ADHD, and SCZ [188]. In this complex scenario, HERVs could represent the link among environmental stimuli, epigenetic remodeling, and biological processes. Indeed, their re-activation in response to external stimuli could determine DNA rearrangements, HERV reinsertions, and HERVs copy number variation, resulting in abnormal HERVs activity that could, in turn, potentially affect the development of the central nervous system.

Due to their regulatory activity, HERVs could affect both the expression of cellular coding genes and the activity of ncRNAs since they can act as enhancers of the transcription [189]. Accordingly, by the analysis of the developing human brain transcriptome, a co-expression of ncRNAs and ASD risk genes [190] has been observed, suggesting that abnormal HERVs expression could contribute to altered neurodevelopment.

Moreover, ncRNA transcribed from HERVs genes can bind cellular microRNA involved in the post-transcriptional regulation of gene expression, since several human microRNAs have high sequence homology with HERVs [191,192]. This process occurs in the regulation of pluripotency of embryonic stem cells when HERV-H long ncRNA (HPAT5, locus 6q27) binds the complementary sequences in the let-7 microRNA family [193,194].

HERVs could interfere with pathways related to the development and function of the nervous system: The human sodium-dependent neutral amino acid transporter type 1 (hASCT1) and hASCT2 have been recognized as the cellular receptors of HERV-W Env [195]. Both receptors play a role in the regulation of glutamatergic transmission in the brain [196], which has been found dysfunctional in schizophrenia and in ASD [197].

In addition, overexpression of the HERV-W *env* gene activated the small conductance Ca2^+^-activated K^+^ channel in human neuroblastoma cells through the cAMP response element (CREB), suggesting that HERV-W *env* gene may interfere with neuronal activity in mental illnesses [198].

**Figure 1:**
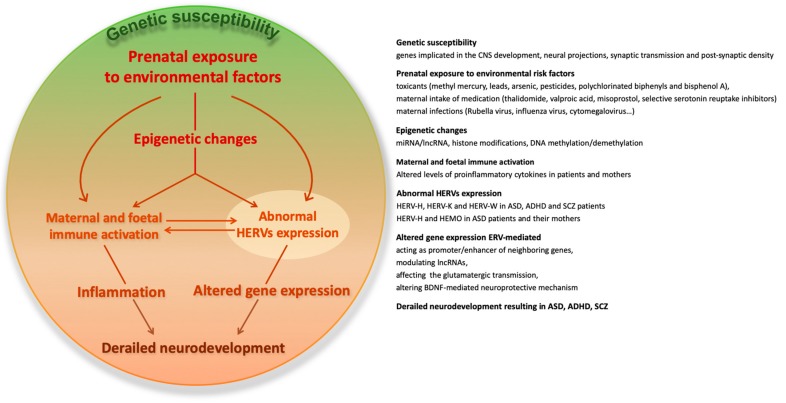
The potential involvement of human endogenous retroviruses (HERVs) in the interaction among genetic vulnerability, environmental risk factors, and immune activation in complex neurodevelopmental disorders.

Moreover, transcriptional activation of HERVs is associated with the development of SCZ; specifically, HERV-W *env* gene regulates the expression of schizophrenia-associated genes, such as the brain-derived neurotrophic factor (BDNF), neurotrophic tyrosine kinase receptor type 2 (NTRK2), and dopamine receptor D3 (DRD3) [175]. BDNF aberrancies have been observed in SCZ [199] and in ASD [200], suggesting a contribution of HERVs in the BDNF-mediated neuroprotective mechanism. In addition, growing evidence suggests that HERVs can shape innate immune response by mechanisms, including the regulation of the expression of neighboring genes and the stimulation of pattern recognition receptors (PRRs): The transactivation of HERVs leads to the release of HERV-derived pathogen-associated molecular patterns (PAMPs), inducing the production of pro-inflammatory effectors, such as IFNs, cytokines, and chemokines [201]. Hence, HERVs could provide continuous triggers to the host immune sensors, since they may be physiologically expressed in few instances [36] and, on the other side, the inflammatory effectors induced by HERVs could, in turn, further increase HERVs activity [57,160,202,203], supporting the hypothesis of an interplay between HERVs and immune system activity (Figure 1).

## 4. Endogenous Retroviruses Activity in Mouse Models of Autism

### 4.1. Mouse Endogenous Retroviruses

The retroelements are not unique in humans, since they are also present in nearly all vertebrates and in some invertebrates [204]. As described in humans, the mouse genome also harbors LINEs and SINEs that are sources of germline mutations via new insertions [205,206]. The mouse genome also contains numerous groups of retrotranspositionally active ERVs that cause the most reported insertional mutations [205]. In mouse, ERVs constitute about 10% of the genome and are typically classified into three classes (class I, II, and III), according to the similarity to the exogenous viral counterpart [12,205]. The majority of ERVs loci exist only as solitary LTRs, as the result of recombination events, and some of ERVs lost coding capability due to mutational degradation, occurred during evolution [205]. Nevertheless, some of them are retrotranspositionally active, leading to germline mutations via new integration events [206]. Particularly, the intracisternal A-particle (IAP) is responsible for the most reported mutations due to new ERVs insertions, with a substantial contribution of the early transposon (ETn)/mouse endogenous proviruses (MusD) ERVs group [206]. IAP sequences belong to class II and are highly abundant in the mouse genome [205]. Although some IAP elements contain an *env* gene, most of them have lost *env* and adopt an intracellular retrotranspositional life cycle [207], resulting in the accumulation of high copy numbers in the genome [208]. Elevated IAP transcripts have been reported during embryogenesis (see Rowe and Trono, 2011, for a comprehensive review) [209] as well as in differentiated tissues, particularly in the thymus [210]. Of note, in lymphoid tissues, somatic insertions of IAP can lead to oncogene or cytokine gene activation [211,212]. Moreover, it is known that IAP can influence the transcriptional profile of nearby genes, providing functional promoter elements and modulating local epigenetic landscape through changes in DNA methylation and histone modifications [213]. In addition to IAPs, the ETn/MusD group is responsible for the next highest number of germline mutations. ETns have no coding capacity, and their retrotransposition is mediated by the coding competent MusD elements [214]. As for IAPs, these elements also encode strictly intracellular virus-like particles since they lack the *env* gene [215]. During the embryogenesis, the expression analysis demonstrated that ETn and MusD are highly transcribed [209], and they are responsive to embryonic transcription factors [216]. Moreover, in mouse embryogenesis, several complex regulatory networks are responsible for the modulation of retroelements, and, in turn, the development is controlled by their temporal and spatial activity. Particularly, IAP elements are carried from the oocyte into early embryos, degraded and then peak again at the blastocyst stage, until the IAP sequences undergo DNA methylation. Conversely, MusD/ETn are highly transcribed in post-implantation embryos [209]. Moreover, two murine *env* genes, each present at a single copy and phylogenetically unrelated to human syncytins, are expressed in the placenta at the level of the syncytiotrophoblast, where they exhibit a fusogenic activity [217]. The conservation of their coding status suggested that their function is most probably similar to that of the human syncytins since mice knockout for either of the two syncytin genes displayed impaired placental trophoblast fusion [218]. Furthermore, more recent data demonstrated the involvement of syncytins in the cell–cell fusion of myoblast in mice [219] and of *ex vivo* human osteoclasts [220] suggesting their crucial role in different host physiologic processes.

### 4.2. Animal Models of Autism

Evidence supporting the involvement of endogenous retroviruses in the course of altered neurodevelopment comes also from studies in preclinical ASD models.

Animal models are crucial tools to deeply understand the human ASD, as they allow the investigation of the pathways and the pathophysiological processes involved, to explore the brain district, mostly inaccessible in humans, and to evaluate the potential translational value of peripheral biomarkers. Several types of ASD animal models have been developed, including those obtained by genetic manipulations, by using behavioral screening of inbred strains of mice to find an ASD-like phenotype and by prenatal exposure to chemicals or infection/inflammation (see Ergaz et al., 2016 for a comprehensive review) [221]. Genetic models consist of mutagenesis or knockout of various isolated genes that are thought to be involved in the pathology of both syndromic and non-syndromic ASD, such as *FMR1* (Fragile X syndrome), *NF1* (Neurofibromatosis type 1), *TSC1* (Tuberous sclerosis), *DHCR7* (Smith–Lemli–Opitz syndrome), *MeCP2* (Rett syndrome), and of genes known to be associated to high risk of ASD, such as *SHANK2*, *CNTNAP2*, *eIF4E*, and transgenic mouse targeting Oxytocin, Vasopressin, Reelin, Dishevelled-1, Sert (serotonin transporter), MAOA (monoamine oxidase A), HOXA1, PTEN, and Neuroligins [221]. On the other side, unknown genetic changes able to induce ASD-like phenotype in animals, in turn, may bring into light similar changes in humans.

Using behavioral screening, the BTBR T+tf/J (BTBR) inbred mice were identified, as they showed several traits relevant to ASD, such as impairments in social and communication domains, reduced cognitive flexibility, and high levels of repetitive behaviors. For this idiopathic ASD model, the inbred C57BL6/J mice have usually been used as a standard control strain [222].

Based on epidemiological studies in humans concerning the association between prenatal infections with increased risk for ASD, other animal models were developed by the prenatal exposure to compounds that stimulate the MIA, such as the poly(I:C) or the LPS to mimic viral and bacterial infection, respectively [223,224]. In rodents, MIA leads to a dysregulation of the immune system in offspring and the acquisition of an autistic-like phenotype until adulthood, and, similar to what is observed in ASD children, IL-6 was supposed to be acting through inhibition of DNA methylation [225]. Notably, by a single poly(I:C) injection, the offspring of the first generation showed an autistic-like phenotype that persists via the paternal lineage, in the second and third generation, without any further intervention [226]. The type of changes that are transmitted through the generations (transgenerational inheritance) are not yet clarified. Nevertheless, a key role played by the immune system could be supposed. Accordingly, an apparent rescue of the behavioral abnormalities was obtained by the administration of neutralizing antibodies against IL-6 and IL-17 [225,227]. Moreover, following MIA induction, several other cytokines, such as TNF-α, IFN-β, and IL-1β, were found overexpressed, but not the anti-inflammatory IL-10 [228].

Based on the clinical evidence, prenatal exposure to VPA has been proposed as a drug-induced model of ASD in rodents [229]. The prenatal exposure to VPA in mice and rats leads to the acquisition of behavioral traits that resemble those observed in autistic patients (decreased social interactions, increased repetitive/stereotypic behaviors, lower sensitivity to pain, impaired sensorimotor gating or eye blink conditioning, increased anxiety, reduced exploratory behavior and abnormally high and longer-lasting fear memories), depending on dose and time of exposure [230].

### 4.3. Evidence of Abnormal ERVs Activity in Mouse Models of Autism

**Figure 2:**
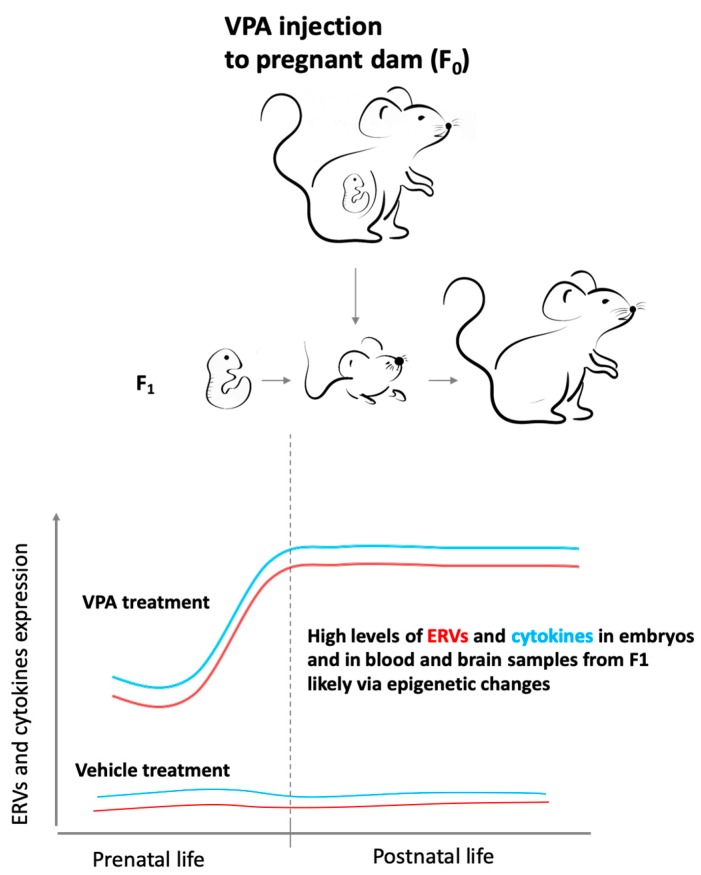
The abnormal endogenous retroviruses (ERVs) and cytokines expression from intrauterine life to adulthood in the first generation (F_1_) prenatally exposed to valproic acid (VPA) could be due to the drug-induced epigenetic changes. ERVs activity (red lines) and cytokines expression (light blue lines) were represented both for VPA- and vehicle-treated mice.

Recently, our group investigated the transcriptional activity of different ERVs, including members of ETn and IAP families, in two models of ASD, the BTBR mice and the CD-1 outbred mice exposed to VPA *in utero* [231]. In both animal models, beginning from intrauterine life and up to adulthood, higher ERVs levels were found in BTBR and VPA-treated animals than in corresponding controls. Particularly, in BTBR mice, the transcriptional activity of ERVs was already altered in whole embryos samples and maintained in both blood and brain samples analyzed at different postnatal ages, suggesting that a long-lasting activation of ERVs could affect brain functions throughout the life span. In the VPA model, ERVs activity was modified immediately after drug administration in embryos, suggesting a direct and rapid effect of VPA. Abnormal expression of ERV has also been found in the offspring (first generation, F_1_) immediately after the birth, both in blood and brain, but high levels, stable until adulthood, were observed only in the brain from VPA-treated mice (Figure 2). In the VPA-induced ASD model, the differences in ERVs expression observed in the two tissues could be attributed to the different cell turnover. In fact, the rapid turnover of blood cells can dilute the VPA effect on the ERVs transcription in these cells, while, since the cellular turnover in the brain is slow/absent, the VPA can induce a permanent increase in ERVs expression, similarly to those found in BTBR mice. Moreover, in both models, the expression of some ERVs families was found to be positively correlated with expression levels of proinflammatory cytokines (IL-1β, IL-6, and TNF-α) and TLR3 and TLR4 in embryos and brain, supporting the hypothesis of the interplay between ERVs activity and immune response [231] (Figure 2).

### 4.4. Recent Findings on the Transgenerational Inheritance of the Abnormal ERVs Expression in a Mouse Model of Autism: the Conceivable Underpinning Mechanisms

Recently, the abnormal expression of ERVs observed in mice prenatally exposed to VPA has also been demonstrated across generations until the third one that lacks direct exposure to the drug, in parallel with the transmission of behavioral alterations [232]. Notably, larger VPA effects on ERVs expression was found in females within the first generation (F_1_) and along maternal lineages in the second and third generation (F_2_ and F_3_, respectively) [232]. The vulnerability of the female sex could be due to the larger epigenetic effect of prenatal VPA exposure reported in female fetal brains, associated with sexually dimorphic methylation of H3K4 induced by VPA [233].

**Figure 3:**
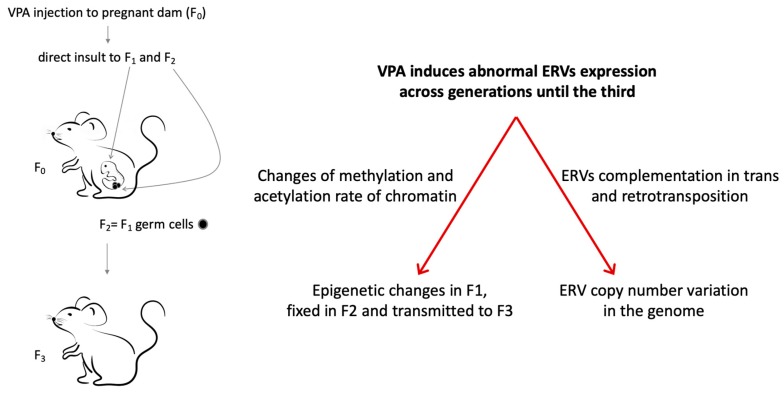
Transgenerational inheritance of altered ERVs activity in the VPA-induced mouse models of autism spectrum disorders (ASD), potentially involved mechanisms. VPA exposure of the pregnant dam (F_0_) leads a direct insult to the fetus (F_1_) and to germ cells that will generate the F_2_ generation, while the F_3_ is the first generation not directly exposed. Transgenerational transmission of abnormal ERVs expression induced by VPA could be due to changes in the epigenetic status or of the ERVs copy number variation in the genome.

The transgenerational transmission of altered ERVs expression could be due to the activity of VPA as a direct HDAC inhibitor, inducing a histone hyperacetylation, but also to its ability to trigger other epigenetic changes, such as histone methylation and DNA demethylation [234]. The administration of the drug during pregnancy could modify the global epigenetic status of the first and also of the second generation (F_2_), present in the embryos of the first generation as germline cells. The VPA-induced epigenetic changes would then be fixed in F_2_ and transmitted to the next generation (F_3_), the first that lacks a direct exposure to the drug [235]. Another mechanism by which ERVs deregulation could be transgenerationally transmitted comprises the acquisition of a newly modified genotype by the increased copy number of ERVs (Figure 3). This hypothesis is in agreement with evidence in the literature, showing that ERVs in mice can cooperate with each other and with non-ERV elements (such as LINEs) by complementation in trans, increasing their intrinsic capability to retrotranspose [206,236] and their proviral copy number. The reintegration of ERVs would lead to the emergence of polymorphisms, as shown in the human genome for HERV-H and HERV-K (HML2) [23,237] without differences in the polymorphism rate between sexes for HERV-K [238]. Moreover, the copy number of HERV-W/MSRV was found to be increased in patients with MS and influenced by gender and disease severity as well as CNV of LINE-1 was found in schizophrenia patients [239,240]. The reintegration of ERVs in the host genome could also contribute to genetic instability and the appearance of chromosome rearrangements, deletions, and duplications according to the detection of CNV and somatic mutation in ASD and SCZ [241,242]. The increase in copy number of ERVs could also explain the more marked effect that prenatal exposure to VPA exerts on ERVs expression in females than males: Oocytes persist long in life while spermatozoids life is short and their high turnover could dilute the effect of the drug.

Transgenerational studies on ASD in preclinical models provide new perspectives in ASD susceptibility, by which autistic traits seem to be inherited in subsequent generations after the first exposure to an insult, thus supporting the view that epigenetic inheritance could play a role in the development and heritability of ASD and more generally in NDDs.

## 5. Conclusions

The exposure to a variety of environmental insults in sensitive time-windows during pregnancy could result in later altered neurodevelopment. The role of HERVs in human embryogenesis, their intrinsic responsiveness to external stimuli, and the interaction with the immune system reinforce the involvement of HERVs in the derailed neurodevelopmental process. However, it is still debated if HERVs are cofactors or epiphenomenon in NDDs, and more efforts are needed to investigate the potentially detrimental effect of HERV-encoded proteins as well as the impact of polymorphisms, unfixed copies, and copy number variation of HERVs in the etiopathogenic processes.

In this complex landscape, the use of animal models for studying NDDs could offer countless advantages: (i) to analyze in greater detail the prenatal phase, that is certainly crucial for the onset of NDDs, (ii) to obtain selective information concerning the brain district, mostly inaccessible in human studies, and (iii) to evaluate potential translational value of peripheral biomarkers. On the other hand, issues of overlapping results between species should be taken into account.

Further studies are needed and could represent a new approach to unravel the etiopathogenesis of NDDs, bearing in mind that the retroelements cannot be appropriately understood through a virologic or genetic approach since they are neither viruses nor physiological genes. Both preclinical models and human studies indicate that the abnormal expression of ERVs could represent a molecular signature of neurodevelopmental disorders.

## Abbreviations

HERVs	Human Endogenous Retroviruses
LTRs	Long Terminal Repeats
LINEs	Long interspersed nuclear elements
RT	Reverse transcriptase
ncRNAs	Non-coding RNA
HEMO	Human endogenous MER34 (medium reiteration-frequency-family-34) ORF
HSV	Herpesviruses
HERV-W/MSRV	Human endogenous retrovirus W/ Multiple Sclerosis-associated retrovirus
EBV	Epstein Barr virus
HHV-6	Human herpesvirus 6
MS	Multiple sclerosis
HTLV-1	Human T-lymphotropic virus-1
HIV-1	Human immunodeficiency virus-1
AIDS	Acquired immune deficiency syndrome
PBMCs	Peripheral blood mononuclear cells
LPS	Lipopolysaccharide
IFN-γ	Interferon-γ
ALS	Amyotrophic lateral sclerosis
TNF-α	Tumor necrosis factor-alpha
IL-1α	Interleukin-1alpha
IL-1β	Interleukin-1β
PHA	Phytohemagglutinin
PMA	Horbol-12-myristate-13-acetate
HDACi	Inhibitors of the histone deacetylase
VPA	Valproic acid
UV	Ultraviolet irradiations
SLE	Systemic lupus erythematosus
RA	Rheumatoid arthritis
TLR4	Toll-Like Receptor 4
ASD	Autism spectrum disorders
ADHD	Attention deficit hyperactivity disorder
SCZ	Schizophrenia
NDDs	Neurodevelopmental disorders
BD	Bipolar disease
CNV	Copy number variation
HDAC1	Inhibitor of histone deacetylase of class I
HDAC2	Inhibitor of histone deacetylase of class II
MIA	Maternal immune activation
MPH	Methylphenidate
BDNF	Brain-derived neurotrophic factor
NTRK2	Neurotrophic tyrosine kinase receptor type 2
DRD3	Dopamine receptor D3
PRRs	Pattern recognition receptors
PAMPs	Pathogen associated molecular patterns
ERVs	Endogenous retroviruses
IAP	Intracisternal A-particle
ETn	Early transposon
MusD	Mouse endogenous proviruses
BTBR	BTBR T+tf/J inbred
F_1_	First generation
F_2_	Second generation
F_3_	Third generation

## References

[1] Lander E.S., Linton L.M., Birren B., Nusbaum C., Zody M.C., Baldwin J., Devon K., Dewar K., Doyle M., FitzHugh W. (2001). International Human Genome Sequencing Consortium. Initial sequencing and analysis of the human genome. Nature.

[2] Feschotte C., Gilbert C. (2012). Endogenous viruses: Insights into viral evolution and impact on host biology. Nat. Rev. Genet..

[3] Kazazian H.H., Moran J.V. (2017). Mobile DNA in Health and Disease. N. Engl. J. Med..

[4] Campos-Sánchez R., Kapusta A., Feschotte C., Chiaromonte F., Makova K.D. (2014). Genomic landscape of human, bat, and ex vivo DNA transposon integrations. Mol. Biol. Evol..

[5] Hancks D.C., Kazazian H.H. (2016). Roles for retrotransposon insertions in human disease. Mob. DNA.

[6] Kassiotis G., Stoye J.P. (2017). Making a virtue of necessity: The pleiotropic role of human endogenous retroviruses in cancer. Philos. Trans. R. Soc. Lond B Biol. Sci..

[7] Ohshima K. (2013). RNA-Mediated Gene Duplication and Retroposons: Retrogenes, LINEs, SINEs, and Sequence Specificity. Int. J. Evol. Biol..

[8] Burns K.H., Boeke J.D. (2012). Human transposon tectonics. Cell.

[9] Belshaw R., Pereira V., Katzourakis A., Talbot G., Paces J., Burt A., Tristem M. (2004). Long-term reinfection of the human genome by endogenous retroviruses. Proc. Natl. Acad. Sci. USA.

[10] Bannert N., Kurth R. (2006). The evolutionary dynamics of human endogenous retroviral families. Annu. Rev. Genomics Hum. Genet..

[11] Bock M., Stoye J.P. (2000). Endogenous retroviruses and the human germline. Curr. Opin. Genet. Dev..

[12] Consortium M.G.S. (2002). Initial sequencing and comparative analysis of the mouse genome. Nature.

[13] Herniou E., Martin J., Miller K., Cook J., Wilkinson M., Tristem M. (1998). Retroviral diversity and distribution in vertebrates. J. Virol..

[14] Jern P., Sperber G.O., Blomberg J. (2006). Divergent patterns of recent retroviral integrations in the human and chimpanzee genomes: Probable transmissions between other primates and chimpanzees. J. Virol..

[15] Nellåker C., Keane T.M., Yalcin B., Wong K., Agam A., Belgard T.G., Flint J., Adams D.J., Frankel W.N., Ponting C.P. (2012). The genomic landscape shaped by selection on transposable elements across 18 mouse strains. Genome Biol..

[16] Paces J., Pavlícek A., Paces V. (2002). HERVd: Database of human endogenous retroviruses. Nucleic Acids Res..

[17] Mayer J., Blomberg J., Seal R.L. (2011). A revised nomenclature for transcribed human endogenous retroviral loci. Mob. DNA.

[18] Tristem M. (2000). Identification and characterization of novel human endogenous retrovirus families by phylogenetic screening of the human genome mapping project database. J. Virol..

[19] Bénit L., Dessen P., Heidmann T. (2001). Identification, phylogeny, and evolution of retroviral elements based on their envelope genes. J. Virol..

[20] Thomas J., Perron H., Feschotte C. (2018). Variation in proviral content among human genomes mediated by LTR recombination. Mob. DNA.

[21] Belshaw R., Katzourakis A., Paces J., Burt A., Tristem M. (2005). High copy number in human endogenous retrovirus families is associated with copying mechanisms in addition to reinfection. Mol. Biol. Evol..

[22] Wildschutte J.H., Williams Z.H., Montesion M., Subramanian R.P., Kidd J.M., Coffin J.M. (2016). Discovery of unfixed endogenous retrovirus insertions in diverse human populations. Proc. Natl. Acad. Sci. USA.

[23] Marchi E., Kanapin A., Magiorkinis G., Belshaw R. (2014). Unfixed endogenous retroviral insertions in the human population. J. Virol..

[24] Coffin J.M., Hughes S.H., Varmus H.E., Coffin J.M., Hughes S.H., Varmus H.E. (1997). The Interactions of Retroviruses and their Hosts. Retroviruses.

[25] Dewannieux M., Heidmann T. (2013). Endogenous retroviruses: Acquisition, amplification and taming of genome invaders. Curr. Opin. Virol..

[26] Montesion M., Williams Z.H., Subramanian R.P., Kuperwasser C., Coffin J.M. (2018). Promoter expression of HERV-K (HML-2) provirus-derived sequences is related to LTR sequence variation and polymorphic transcription factor binding sites. Retrovirology.

[27] Jern P., Coffin J.M. (2008). Effects of retroviruses on host genome function. Annu. Rev. Genet..

[28] Rebollo R., Romanish M.T., Mager D.L. (2012). Transposable elements: An abundant and natural source of regulatory sequences for host genes. Annu. Rev. Genet..

[29] Frank J.A., Feschotte C. (2017). Co-option of endogenous viral sequences for host cell function. Curr. Opin. Virol..

[30] Chuong E.B., Elde N.C., Feschotte C. (2017). Regulatory activities of transposable elements: From conflicts to benefits. Nat. Rev. Genet..

[31] Leboyer M., Tamouza R., Charron D., Faucard R., Perron H. (2013). Human endogenous retrovirus type W (HERV-W) in schizophrenia: A new avenue of research at the gene-environment interface. World J. Biol. Psychiatry.

[32] Matteucci C., Balestrieri E., Argaw-Denboba A., Sinibaldi-Vallebona P. (2018). Human endogenous retroviruses role in cancer cell stemness. Semin. Cancer Biol..

[33] Küry P., Nath A., Créange A., Dolei A., Marche P., Gold J., Giovannoni G., Hartung H.P., Perron H. (2018). Human Endogenous Retroviruses in Neurological Diseases. Trends Mol. Med..

[34] Ehlhardt S., Seifert M., Schneider J., Ojak A., Zang K.D., Mehraein Y. (2006). Human endogenous retrovirus HERV-K(HML-2) Rec expression and transcriptional activities in normal and rheumatoid arthritis synovia. J. Rheumatol..

[35] Schmitt K., Heyne K., Roemer K., Meese E., Mayer J. (2015). HERV-K(HML-2) rec and np9 transcripts not restricted to disease but present in many normal human tissues. Mob. DNA.

[36] Balestrieri E., Pica F., Matteucci C., Zenobi R., Sorrentino R., Argaw-Denboba A., Cipriani C., Bucci I., Sinibaldi-Vallebona P. (2015). Transcriptional activity of human endogenous retroviruses in human peripheral blood mononuclear cells. Biomed. Res. Int..

[37] Buzdin A., Kovalskaya-Alexandrova E., Gogvadze E., Sverdlov E. (2006). At least 50% of human-specific HERV-K (HML-2) long terminal repeats serve in vivo as active promoters for host nonrepetitive DNA transcription. J. Virol.

[38] Chuong E.B., Rumi M.A., Soares M.J., Baker J.C. (2013). Endogenous retroviruses function as species-specific enhancer elements in the placenta. Nat. Genet..

[39] Suntsova M., Garazha A., Ivanova A., Kaminsky D., Zhavoronkov A., Buzdin A. (2015). Molecular functions of human endogenous retroviruses in health and disease. Cell. Mol. Life Sci..

[40] Schumann G.G., Gogvadze E.V., Osanai-Futahashi M., Kuroki A., Münk C., Fujiwara H., Ivics Z., Buzdin A.A. (2010). Unique functions of repetitive transcriptomes. Int. Rev. Cell Mol. Biol..

[41] Wang J., Xie G., Singh M., Ghanbarian A.T., Raskó T., Szvetnik A., Cai H., Besser D., Prigione A., Fuchs N.V. (2014). Primate-specific endogenous retrovirus-driven transcription defines naive-like stem cells. Nature.

[42] Hughes J.F., Coffin J.M. (2004). Human endogenous retrovirus K solo-LTR formation and insertional polymorphisms: Implications for human and viral evolution. Proc. Natl. Acad. Sci. USA.

[43] Trombetta B., Fantini G., D’Atanasio E., Sellitto D., Cruciani F. (2016). Evidence of extensive non-allelic gene conversion among LTR elements in the human genome. Sci. Rep..

[44] Dunn C.A., Medstrand P., Mager D.L. (2003). An endogenous retroviral long terminal repeat is the dominant promoter for human beta1,3-galactosyltransferase 5 in the colon. Proc. Natl. Acad. Sci. USA.

[45] Bièche I., Laurent A., Laurendeau I., Duret L., Giovangrandi Y., Frendo J.L., Olivi M., Fausser J.L., Evain-Brion D., Vidaud M. (2003). Placenta-specific INSL4 expression is mediated by a human endogenous retrovirus element. Biol. Reprod..

[46] Ting C.N., Rosenberg M.P., Snow C.M., Samuelson L.C., Meisler M.H. (1992). Endogenous retroviral sequences are required for tissue-specific expression of a human salivary amylase gene. Genes Dev..

[47] Grow E.J., Flynn R.A., Chavez S.L., Bayless N.L., Wossidlo M., Wesche D.J., Martin L., Ware C.B., Blish C.A., Chang H.Y. (2015). Intrinsic retroviral reactivation in human preimplantation embryos and pluripotent cells. Nature.

[48] Glinsky G.V. (2015). Transposable Elements and DNA Methylation Create in Embryonic Stem Cells Human-Specific Regulatory Sequences Associated with Distal Enhancers and Noncoding RNAs. Genome Biol. Evol..

[49] Gemmell P., Hein J., Katzourakis A. (2016). Phylogenetic Analysis Reveals That ERVs “Die Young” but HERV-H Is Unusually Conserved. PLoSComput. Biol..

[50] Heidmann O., Béguin A., Paternina J., Berthier R., Deloger M., Bawa O., Heidmann T. (2017). HEMO, an ancestral endogenous retroviral envelope protein shed in the blood of pregnant women and expressed in pluripotent stem cells and tumors. Proc. Natl. Acad. Sci. USA.

[51] Mi S., Lee X., Li X., Veldman G.M., Finnerty H., Racie L., LaVallie E., Tang X.Y., Edouard P., Howes S. (2000). Syncytin is a captive retroviral envelope protein involved in human placental morphogenesis. Nature.

[52] Chuong E.B., Elde N.C., Feschotte C. (2016). Regulatory evolution of innate immunity through co-option of endogenous retroviruses. Science.

[53] Blaise S., de Parseval N., Bénit L., Heidmann T. (2003). Genomewide screening for fusogenic human endogenous retrovirus envelopes identifies syncytin 2, a gene conserved on primate evolution. Proc. Natl. Acad. Sci. USA.

[54] Frendo J.L., Olivier D., Cheynet V., Blond J.L., Bouton O., Vidaud M., Rabreau M., Evain-Brion D., Mallet F. (2003). Direct involvement of HERV-W Env glycoprotein in human trophoblast cell fusion and differentiation. Mol. Cell. Biol..

[55] Dunlap K.A., Palmarini M., Varela M., Burghardt R.C., Hayashi K., Farmer J.L., Spencer T.E. (2006). Endogenous retroviruses regulate periimplantation placental growth and differentiation. Proc. Natl. Acad. Sci. USA.

[56] Engel M.E., Hiebert S.W. (2010). The enemy within: Dormant retroviruses awaken. Nat. Med..

[57] Gröger V., Cynis H. (2018). Human Endogenous Retroviruses and Their Putative Role in the Development of Autoimmune Disorders Such as Multiple Sclerosis. Front. Microbiol..

[58] Grandi N., Tramontano E. (2018). HERV Envelope Proteins: Physiological Role and Pathogenic Potential in Cancer and Autoimmunity. Front. Microbiol..

[59] Chen J., Foroozesh M., Qin Z. (2019). Transactivation of human endogenous retroviruses by tumor viruses and their functions in virus-associated malignancies. Oncogenesis.

[60] Nellåker C., Yao Y., Jones-Brando L., Mallet F., Yolken R.H., Karlsson H. (2006). Transactivation of elements in the human endogenous retrovirus W family by viral infection. Retrovirology.

[61] Ruprecht K., Obojes K., Wengel V., Gronen F., Kim K.S., Perron H., Schneider-Schaulies J., Rieckmann P. (2006). Regulation of human endogenous retrovirus W protein expression by herpes simplex virus type 1: Implications for multiple sclerosis. J. Neurovirol..

[62] Mameli G., Poddighe L., Mei A., Uleri E., Sotgiu S., Serra C., Manetti R., Dolei A. (2012). Expression and activation by Epstein Barr virus of human endogenous Retroviruses-W in blood cells and astrocytes: Inference for multiple sclerosis. PLoS ONE.

[63] Hsiao F.C., Lin M., Tai A., Chen G., Huber B.T. (2006). Cutting edge: Epstein-Barr virus transactivates the HERV-K18 superantigen by docking to the human complement receptor 2 (CD21) on primary B cells. J. Immunol..

[64] Leung A., Trac C., Kato H., Costello K.R., Chen Z., Natarajan R., Schones D.E. (2018). LTRs activated by Epstein-Barr virus-induced transformation of B cells alter the transcriptome. Genome Res..

[65] Dai L., Del Valle L., Miley W., Whitby D., Ochoa A.C., Flemington E.K., Qin Z. (2018). Transactivation of human endogenous retrovirus K (HERV-K) by KSHV promotes Kaposi’s sarcoma development. Oncogene.

[66] Charvet B., Reynaud J.M., Gourru-Lesimple G., Perron H., Marche P.N., Horvat B. (2018). Induction of Proinflammatory Multiple Sclerosis-Associated Retrovirus Envelope Protein by Human Herpesvirus-6A and CD46 Receptor Engagement. Front. Immunol..

[67] Wang W., Jovel J., Halloran B., Wine E., Patterson J., Ford G., O’Keefe S., Meng B., Song D., Zhang Y. (2015). Metagenomic analysis of microbiome in colon tissue from subjects with inflammatory bowel diseases reveals interplay of viruses and bacteria. Inflamm. Bowel Dis..

[68] Liu C., Liu L., Wang X., Liu Y., Wang M., Zhu F. (2017). HBV X Protein induces overexpression of HERV-W env through NF-κB in HepG2 cells. Virus Genes.

[69] Toufaily C., Landry S., Leib-Mosch C., Rassart E., Barbeau B. (2011). Activation of LTRs from different human endogenous retrovirus (HERV) families by the HTLV-1 tax protein and T-cell activators. Viruses.

[70] Contreras-Galindo R., Kaplan M.H., Markovitz D.M., Lorenzo E., Yamamura Y. (2006). Detection of HERV-K(HML-2) viral RNA in plasma of HIV type 1-infected individuals. AIDS Res. Hum. Retrovir..

[71] Young G.R., Terry S.N., Manganaro L., Cuesta-Dominguez A., Deikus G., Bernal-Rubio D., Campisi L., Fernandez-Sesma A., Sebra R., Simon V. (2017). HIV-1 Infection of Primary CD4+ T Cells Regulates the Expression of Specific Human Endogenous Retrovirus HERV-K (HML-2) Elements. J. Virol..

[72] Gonzalez-Hernandez M.J., Cavalcoli J.D., Sartor M.A., Contreras-Galindo R., Meng F., Dai M., Dube D., Saha A.K., Gitlin S.D., Omenn G.S. (2014). Regulation of the human endogenous retrovirus K (HML-2) transcriptome by the HIV-1 Tat protein. J. Virol..

[73] Uleri E., Mei A., Mameli G., Poddighe L., Serra C., Dolei A. (2014). HIV Tat acts on endogenous retroviruses of the W family and this occurs via Toll-like receptor 4: Inference for neuroAIDS. AIDS.

[74] Li F., Nellåker C., Sabunciyan S., Yolken R.H., Jones-Brando L., Johansson A.S., Owe-Larsson B., Karlsson H. (2014). Transcriptional derepression of the ERVWE1 locus following influenza A virus infection. J. Virol..

[75] Frank O., Jones-Brando L., Leib-Mosch C., Yolken R., Seifarth W. (2006). Altered transcriptional activity of human endogenous retroviruses in neuroepithelial cells after infection with Toxoplasma gondii. J. Infect. Dis..

[76] Young G.R., Mavrommatis B., Kassiotis G. (2014). Microarray analysis reveals global modulation of endogenous retroelement transcription by microbes. Retrovirology.

[77] Mommert M., Tabone O., Oriol G., Cerrato E., Guichard A., Naville M., Fournier P., Volff J.N., Pachot A., Monneret G. (2018). LTR-retrotransposon transcriptome modulation in response to endotoxin-induced stress in PBMCs. BMC Genomics.

[78] Manghera M., Ferguson-Parry J., Lin R., Douville R.N. (2016). NF-κB and IRF1 Induce Endogenous Retrovirus K Expression via Interferon-Stimulated Response Elements in Its 5’ Long Terminal Repeat. J. Virol..

[79] Katsumata K., Ikeda H., Sato M., Ishizu A., Kawarada Y., Kato H., Wakisaka A., Koike T., Yoshiki T. (1999). Cytokine regulation of env gene expression of human endogenous Retrovirus-R in human vascular endothelial cells. Clin. Immunol..

[80] Mueller O., Moore D.W., Giovannucci J., Etter A.R., Peterson E.M., Mudge A., Liu Y. (2018). Expression of Human Endogenous Retroviruses in Peripheral Leukocytes During the Menstrual Cycle Suggests Coordinated Hormonal Regulation. AIDS Res. Hum. Retrovir..

[81] Wang-Johanning F., Frost A.R., Jian B., Epp L., Lu D.W., Johanning G.L. (2003). Quantitation of HERV-K env gene expression and splicing in human breast cancer. Oncogene.

[82] Buslei R., Strissel P.L., Henke C., Schey R., Lang N., Ruebner M., Stolt C.C., Fabry B., Buchfelder M., Strick R. (2015). Activation and regulation of endogenous retroviral genes in the human pituitary gland and related endocrine tumours. Neuropathol. Appl. Neurobiol..

[83] Ogasawara H., Naito T., Kaneko H., Hishikawa T., Sekigawa I., Hashimoto H., Kaneko Y., Yamamoto N., Maruyama N., Yamamoto N. (2001). Quantitative analyses of messenger RNA of human endogenous retrovirus in patients with systemic lupus erythematosus. J. Rheumatol..

[84] Kelleher C.A., Wilkinson D.A., Freeman J.D., Mager D.L., Gelfand E.W. (1996). Expression of novel-transposon-containing mRNAs in human T cells. J. Gen. Virol..

[85] Johnston J.B., Silva C., Holden J., Warren K.G., Clark A.W., Power C. (2001). Monocyte activation and differentiation augment human endogenous retrovirus expression: Implications for inflammatory brain diseases. Ann. Neurol..

[86] Bollati V., Favero C., Albetti B., Tarantini L., Moroni A., Byun H.M., Motta V., Conti D.M., Tirelli A.S., Vigna L. (2014). Nutrients intake is associated with DNA methylation of candidate inflammatory genes in a population of obese subjects. Nutrients.

[87] Liu M., Ohtani H., Zhou W., Ørskov A.D., Charlet J., Zhang Y.W., Shen H., Baylin S.B., Liang G., Grønbæk K. (2016). Vitamin C increases viral mimicry induced by 5-aza-2′-deoxycytidine. Proc. Natl. Acad. Sci. USA.

[88] Serafino A., Balestrieri E., Pierimarchi P., Matteucci C., Moroni G., Oricchio E., Rasi G., Mastino A., Spadafora C., Garaci E. (2009). The activation of human endogenous retrovirus K (HERV-K) is implicated in melanoma cell malignant transformation. Exp. Cell. Res..

[89] Argaw-Denboba A., Balestrieri E., Serafino A., Cipriani C., Bucci I., Sorrentino R., Sciamanna I., Gambacurta A., Sinibaldi-Vallebona P., Matteucci C. (2017). HERV-K activation is strictly required to sustain CD133+ melanoma cells with stemness features. J. Exp. Clin. Cancer Res..

[90] Balestrieri E., Argaw-Denboba A., Gambacurta A., Cipriani C., Bei R., Serafino A., Sinibaldi-Vallebona P., Matteucci C. (2018). Human Endogenous Retrovirus K in the Crosstalk Between Cancer Cells Microenvironment and Plasticity: A New Perspective for Combination Therapy. Front. Microbiol..

[91] Díaz-Carballo D., Acikelli A.H., Klein J., Jastrow H., Dammann P., Wyganowski T., Guemues C., Gustmann S., Bardenheuer W., Malak S. (2015). Therapeutic potential of antiviral drugs targeting chemorefractory colorectal adenocarcinoma cells overexpressing endogenous retroviral elements. J. Exp. Clin. Cancer Res..

[92] Diem O., Schäffner M., Seifarth W., Leib-Mösch C. (2012). Influence of antipsychotic drugs on human endogenous retrovirus (HERV) transcription in brain cells. PLoS ONE.

[93] White C.H., Beliakova-Bethell N., Lada S.M., Breen M.S., Hurst T.P., Spina C.A., Richman D.D., Frater J., Magiorkinis G., Woelk C.H. (2018). Transcriptional Modulation of Human Endogenous Retroviruses in Primary CD4+ T Cells Following Vorinostat Treatment. Front. Immunol..

[94] Conti A., Rota F., Ragni E., Favero C., Motta V., Lazzari L., Bollati V., Fustinoni S., Dieci G. (2016). Hydroquinoneinduces DNA hypomethylation-independentoverexpression of retroelements in human leukemia and hematopoieticstemcells. Biochem. Biophys. Res. Commun..

[95] Schanab O., Humer J., Gleiss A., Mikula M., Sturlan S., Grunt S., Okamoto I., Muster T., Pehamberger H., Waltenberger A. (2011). Expression of human endogenous retrovirus K is stimulated by ultraviolet radiation in melanoma. Pigment Cell Melanoma Res..

[96] Wu Z., Mei X., Zhao D., Sun Y., Song J., Pan W., Shi W. (2015). DNA methylation modulates HERV-E expression in CD4+ T cells from systemic lupus erythematosus patients. J. Dermatol. Sci..

[97] Sauter M., Schommer S., Kremmer E., Remberger K., Dölken G., Lemm I., Buck M., Best B., Neumann-Haefelin D., Mueller-Lantzsch N. (1995). Human endogenous retrovirus K10: Expression of Gag protein and detection of antibodies in patients with seminomas. J. Virol..

[98] Wang-Johanning F., Li M., Esteva F.J., Hess K.R., Yin B., Rycaj K., Plummer J.B., Garza J.G., Ambs S., Johanning G.L. (2014). Human endogenous retrovirus type K antibodies and mRNA as serum biomarkers of early-stage breast cancer. Int. J. Cancer.

[99] Giovinazzo A., Balestrieri E., Petrone V., Argaw-Denboba A., Cipriani C., Miele M.T., Grelli S., Sinibaldi-Vallebona P., Matteucci C. (2019). The concomitant expression of human endogenous retroviruses and embryonic genes in cancer cells under microenvironmental changes is a potential target for antiretroviral drugs. Cancer Microenviron..

[100] Perron H., Dougier-Reynaud H.L., Lomparski C., Popa I., Firouzi R., Bertrand J.B., Marusic S., Portoukalian J., Jouvin-Marche E., Villiers C.L. (2013). Human endogenous retrovirus protein activates innate immunity and promotes experimental allergic encephalomyelitis in mice. PLoS ONE.

[101] Curtin F., Perron H., Kromminga A., Porchet H., Lang A.B. (2015). Preclinical and early clinical development of GNbAC1, a humanized IgG4 monoclonal antibody targeting endogenous retroviral MSRV-Env protein. mAbs.

[102] Nelson P.N., Roden D., Nevill A., Freimanis G.L., Trela M., Ejtehadi H.D., Bowman S., Axford J., Veitch A.M., Tugnet N. (2014). Rheumatoid arthritis is associated with IgG antibodies to human endogenous retrovirus gag matrix: A potential pathogenic mechanism of disease?. J. Rheumatol..

[103] Bendiksen S., Martinez-Zubiavrra I., Tümmler C., Knutsen G., Elvenes J., Olsen E., Olsen R., Moens U. (2014). Human endogenous retrovirus W activity in cartilage of osteoarthritis patients. Biomed. Res. Int..

[104] Marguerat S., Wang W.Y., Todd J.A., Conrad B. (2004). Association of human endogenous retrovirus K-18 polymorphisms with type 1 diabetes. Diabetes.

[105] Levet S., Medina J., Joanou J., Demolder A., Queruel N., Réant K., Normand M., Seffals M., Dimier J., Germi R. (2017). An ancestral retroviral protein identified as a therapeutic target in type-1 diabetes. JCI Insight.

[106] Moreno-De-Luca A., Myers S.M., Challman T.D., Moreno-De-Luca D., Evans D.W., Ledbetter D.H. (2013). Developmental brain dysfunction: Revival and expansion of old concepts based on new genetic evidence. Lancet Neurol..

[107] Ploeger A., Raijmakers M.E., van der Maas H.L., Galis F. (2010). The association between autism and errors in early embryogenesis: What is the causal mechanism?. Biol. Psychiatry.

[108] American Psychiatric Association (2013). Diagnostic and Statistical Manual of Mental Disorders.

[109] Cannon T.D., van Erp T.G., Bearden C.E., Loewy R., Thompson P., Toga A.W., Huttunen M.O., Keshavan M.S., Seidman L.J., Tsuang M.T. (2003). Early and late neurodevelopmental influences in the prodrome to schizophrenia: Contributions of genes, environment, and their interactions. Schizophr. Bull..

[110] Rapoport J., Chavez A., Greenstein D., Addington A., Gogtay N. (2009). Autism spectrum disorders and childhood-onset schizophrenia: Clinical and biological contributions to a relation revisited. J. Am. Acad. Child Adolesc. Psychiatry.

[111] Park M.T.M., Raznahan A., Shaw P., Gogtay N., Lerch J.P., Chakravarty M.M. (2018). Neuroanatomical phenotypes in mental illness: Identifying convergent and divergent cortical phenotypes across autism, ADHD and schizophrenia. J. Psychiatry Neurosci..

[112] Septier M., Peyre H., Amsellem F., Beggiato A., Maruani A., Poumeyreau M., Amestoy A., Scheid I., Gaman A., Bolognani F. (2019). Increased risk of ADHD in families with ASD. Eur. Child Adolesc. Psychiatry.

[113] Simonoff E., Pickles A., Charman T., Chandler S., Loucas T., Baird G. (2008). Psychiatric disorders in children with autism spectrum disorders: Prevalence, comorbidity, and associated factors in a population-derived sample. J. Am. Acad. Child Adolesc. Psychiatry.

[114] Jokiranta-Olkoniemi E., Cheslack-Postava K., Sucksdorff D., Suominen A., Gyllenberg D., Chudal R., Leivonen S., Gissler M., Brown A.S., Sourander A. (2016). Risk of Psychiatric and Neurodevelopmental Disorders Among Siblings of Probands with Autism Spectrum Disorders. JAMA Psychiatry.

[115] Guilmatre A., Dubourg C., Mosca A.L., Legallic S., Goldenberg A., Drouin-Garraud V., Layet V., Rosier A., Briault S., Bonnet-Brilhault F. (2009). Recurrent rearrangements in synaptic and neurodevelopmental genes and shared biologic pathways in schizophrenia, autism, and mental retardation. Arch. Gen. Psychiatry.

[116] Cross-Disorder Group of the Psychiatric Genomics Consortium (2013). Identification of risk loci with shared effects on five major psychiatric disorders: A genome-wide analysis. Lancet.

[117] Anttila V., Bulik-Sullivan B., Finucane H.K., Walters R.K., Bras J., Duncan L., Escott-Price V., Falcone G.J., Gormley P., Brainstorm Consortium (2018). Analysis of shared heritability in common disorders of the brain. Science.

[118] Lotan A., Fenckova M., Bralten J., Alttoa A., Dixson L., Williams R.W., van der Voet M. (2014). Neuroinformatic analyses of common and distinct genetic components associated with major neuropsychiatric disorders. Front. Neurosci..

[119] Liu X., Li Z., Fan C., Zhang D., Chen J. (2017). Genetics implicate common mechanisms in autism and schizophrenia: Synaptic activity and immunity. J. Med. Genet..

[120] Glessner J.T., Li J., Wang D., March M., Lima L., Desai A., Hadley D., Kao C., Gur R.E., Cohen N. (2017). Copy number variation meta-analysis reveals a novel duplication at 9p24 associated with multiple neurodevelopmental disorders. Genome Med..

[121] Brown A.S., Derkits E.J. (2010). Prenatal infection and schizophrenia: A review of epidemiologic and translational studies. Am. J. Psychiatry.

[122] Heyer D.B., Meredith R.M. (2017). Environmental toxicology: Sensitive periods of development and neurodevelopmental disorders. Neurotoxicology.

[123] Miodovnik A., Engel S.M., Zhu C., Ye X., Soorya L.V., Silva M.J., Calafat A.M., Wolff M.S. (2011). Endocrine disruptors and childhood social impairment. Neurotoxicology.

[124] Christensen J., Grønborg T.K., Sørensen M.J., Schendel D., Parner E.T., Pedersen L.H., Vestergaard M. (2013). Prenatal valproate exposure and risk of autism spectrum disorders and childhood autism. JAMA.

[125] Bromley R.L., Mawer G.E., Briggs M., Cheyne C., Clayton-Smith J., García-Fiñana M., Kneen R., Lucas S.B., Shallcross R., Baker G.A. (2013). The prevalence of neurodevelopmental disorders in children prenatally exposed to antiepileptic drugs. J. Neurol. Neurosurg Psychiatry.

[126] Soontornpun A., Choovanichvong T., Tongsong T. (2018). Pregnancy outcomes among women with epilepsy: A retrospective cohort study. Epilepsy Behav..

[127] Ornoy A. (2009). Valproic acid in pregnancy: How much are we endangering the embryo and fetus?. Reprod. Toxicol..

[128] Kolozsi E., Mackenzie R.N., Roullet F., deCatanzaro D., Foster J.A. (2009). Prenatal exposure to valproic acid leads to reduced expression of synaptic adhesion molecule neuroligin 3 in mice. Neuroscience.

[129] Kazlauskas N., Campolongo M., Lucchina L., Zappala C., Depino A.M. (2016). Postnatal behavioral and inflammatory alterations in female pups prenatally exposed to valproic acid. Psychoneuroendocrinology..

[130] Manent J.B., Jorquera I., Mazzucchelli I., Depaulis A., Perucca E., Ben-Ari Y., Represa A. (2007). Fetal exposure to GABA-acting antiepileptic drugs generates hippocampal and cortical dysplasias. Epilepsia.

[131] Schneider T., Roman A., Basta-Kaim A., Kubera M., Budziszewska B., Schneider K., Przewłocki R. (2008). Gender-specific behavioral and immunological alterations in an animal model of autism induced by prenatal exposure to valproic acid. Psychoneuroendocrinology.

[132] Fukuchi M., Nii T., Ishimaru N., Minamino A., Hara D., Takasaki I., Tabuchi A., Tsuda M. (2009). Valproic acid induces up- or down-regulation of gene expression responsible for the neuronal excitation and inhibition in rat cortical neurons through its epigenetic actions. Neurosci. Res..

[133] Barrett C.E., Hennessey T.M., Gordon K.M., Ryan S.J., McNair M.L., Ressler K.J., Rainnie D.G. (2017). Developmental disruption of amygdala transcriptome and socioemotional behavior in rats exposed to valproic acid prenatally. Mol. Autism.

[134] Kawanai T., Ago Y., Watanabe R., Inoue A., Taruta A., Onaka Y., Hasebe S., Hashimoto H., Matsuda T., Takuma K. (2016). Prenatal Exposure to Histone Deacetylase Inhibitors Affects Gene Expression of Autism-Related Molecules and Delays Neuronal Maturation. Neurochem. Res..

[135] Choi C.S., Gonzales E.L., Kim K.C., Yang S.M., Kim J.W., Mabunga D.F., Cheong J.H., Han S.H., Bahn G.H., Shin C.Y. (2016). The transgenerational inheritance of autism-like phenotypes in mice exposed to valproic acid during pregnancy. Sci. Rep..

[136] Chess S. (1971). Autism in children with congenital rubella. J. Autism Child. Schizophr..

[137] Lundstrom R., Ahnsjo S. (1962). Mental development following maternal rubella:A follow-up study of children born in 1951–1952. Acta Paediatr..

[138] Brown A.S., Susser E.S. (2002). In utero infection and adult schizophrenia. Ment. Retard. Dev. Disabil. Res. Rev..

[139] Brown A.S., Begg M.D., Gravenstein S., Schaefer C.A., Wyatt R.J., Bresnahan M., Babulas V.P., Susser E.S. (2004). Serologic evidence of prenatal influenza in the etiology of schizophrenia. Arch. Gen. Psychiatry.

[140] Mann J.R., McDermott S. (2011). Are maternal genitourinary infection and pre-eclampsia associated with ADHD in school-aged children?. J. Atten. Disord..

[141] Werenberg Dreier J., Nybo Andersen A.M., Hvolby A., Garne E., Kragh Andersen P., Berg-Beckhoff G. (2016). Fever and infections in pregnancy and risk of attention deficit/hyperactivity disorder in the offspring. J. Child Psychol. Psychiatry.

[142] Atladóttir H.O., Thorsen P., Østergaard L., Schendel D.E., Lemcke S., Abdallah M., Parner E.T. (2010). Maternal infection requiring hospitalization during pregnancy and autism spectrum disorders. J. Autism Dev. Disord..

[143] Maeyama K., Tomioka K., Nagase H., Yoshioka M., Takagi Y., Kato T., Mizobuchi M., Kitayama S., Takada S., Nagai M. (2018). Congenital Cytomegalovirus Infection in Children with Autism Spectrum Disorder: Systematic Review and Meta-Analysis. J. Autism Dev. Disord..

[144] Lintas C., Altieri L., Lombardi F., Sacco R., Persico A.M. (2010). Association of autism with polyomavirus infection in postmortem brains. J. Neurovirol..

[145] Atladóttir H.Ó., Henriksen T.B., Schendel D.E., Parner E.T. (2012). Autism after infection, febrile episodes, and antibiotic use during pregnancy: An exploratory study. Pediatrics.

[146] Shi L., Fatemi S.H., Sidwell R.W., Patterson P.H. (2003). Maternal influenza infection causes marked behavioral and pharmacological changes in the offspring. J. Neurosci..

[147] Knuesel I., Chicha L., Britschgi M., Schobel S.A., Bodmer M., Hellings J.A., Toovey S., Prinssen E.P. (2014). Maternal immune activation and abnormal brain development across CNS disorders. Nat. Rev. Neurol..

[148] Estes M.L., McAllister A.K. (2016). Maternal immune activation: Implications for neuropsychiatric disorders. Science.

[149] Gilmore J., Jarskog L.F., Vadlamudi S. (2005). Maternal poly I:C exposure during pregnancy regulates TNF alpha, BDNF, and NGF expression in neonatal brain and the maternal-fetal unit of the rat. J. Neuroimmunol..

[150] Urakubo A., Jarskog L.F., Lieberman J.A., Gilmore J.H. (2001). Prenatal exposure to maternal infection alters cytokine expression in the placenta, amniotic fluid, and fetal brain. Schizophr. Res..

[151] Zuckerman L., Weiner I. (2005). Maternal immune activation leads to behavioral and pharmacological changes in the adult offspring. J. Psychiatr Res..

[152] Meyer U., Feldon J. (2009). Neural basis of psychosis-related behaviour in the infection model of schizophrenia. Behav. Brain Res..

[153] Shi L., Smith S.E., Malkova N., Tse D., Su Y., Patterson P.H. (2009). Activation of the maternal immune system alters cerebellar development in the offspring. Brain Behav. Immun..

[154] Goines P.E., Croen L.A., Braunschweig D., Yoshida C.K., Grether J., Hansen R., Kharrazi M., Ashwood P., Van de Water J. (2011). Increased midgestational IFN-γ, IL-4 and IL-5 in women bearing a child with autism: A case-control study. Mol. Autism.

[155] Abdallah M.W., Larsen N., Grove J., Nørgaard-Pedersen B., Thorsen P., Mortensen E.L., Hougaard D.M. (2013). Amniotic fluid inflammatory cytokines: Potential markers of immunologic dysfunction in autism spectrum disorders. World J. Biol. Psychiatry.

[156] Jones K.L., Croen L.A., Yoshida C.K., Heuer L., Hansen R., Zerbo O., DeLorenze G.N., Kharrazi M., Yolken R., Ashwood P. (2017). Autism with intellectual disability is associated with increased levels of maternal cytokines and chemokines during gestation. Mol. Psychiatry.

[157] Meyer U., Feldon J., Dammann O. (2011). Schizophrenia and autism: Both shared and disorder-specific pathogenesis via perinatal inflammation?. Pediatr. Res..

[158] Hsiao E.Y., McBride S.W., Chow J., Mazmanian S.K., Patterson P.H. (2012). Modeling an autism risk factor in mice leads to permanent immune dysregulation. Proc. Natl. Acad. Sci. USA.

[159] Masi A., Glozier N., Dale R., Guastella A.J. (2017). The Immune System, Cytokines, and Biomarkers in Autism Spectrum Disorder. Neurosci. Bull..

[160] Balestrieri E., Cipriani C., Matteucci C., Benvenuto A., Coniglio A., Argaw-Denboba A., Toschi N., Bucci I., Miele M.T., Grelli S. (2019). Children with Autism Spectrum Disorder and Their Mothers Share Abnormal Expression of Selected Endogenous Retroviruses Families and Cytokines. Front. Immunol..

[161] Verlaet A.A., Noriega D.B., Hermans N., Savelkoul H.F. (2014). Nutrition, immunological mechanisms and dietary immunomodulation in ADHD. Eur. Child Adolesc. Psychiatry.

[162] Instanes J.T., Halmøy A., Engeland A., Haavik J., Furu K., Klungsøyr K. (2017). Attention-Deficit/Hyperactivity Disorder in Offspring of Mothers with Inflammatory and Immune System Diseases. Biol. Psychiatry.

[163] Miller B.J., Buckley P., Seabolt W., Mellor A., Kirkpatrick B. (2011). Meta-analysis of cytokine alterations in schizophrenia: Clinical status and antipsychotic effects. Biol. Psychiatry.

[164] Upthegrove R., Manzanares-Teson N., Barnes N.M. (2014). Cytokine function in medication-naive first episode psychosis: A systematic review and meta-analysis. Schizophr. Res..

[165] Khoury R., Nasrallah H.A. (2018). Inflammatory biomarkers in individuals at clinical high risk for psychosis (CHR-P): State or trait?. Schizophr. Res..

[166] Goldsmith D.R., Rapaport M.H., Miller B.J. (2016). A meta-analysis of blood cytokine network alterations in psychiatric patients: Comparisons between schizophrenia, bipolar disorder and depression. Mol. Psychiatry.

[167] Balestrieri E., Arpino C., Matteucci C., Sorrentino R., Pica F., Alessandrelli R., Coniglio A., Curatolo P., Rezza G., Macciardi F. (2012). HERVs expression in Autism Spectrum Disorders. PLoS ONE.

[168] Balestrieri E., Pitzianti M., Matteucci C., D’Agati E., Sorrentino R., Baratta A., Caterina R., Zenobi R., Curatolo P., Garaci E. (2014). Human endogenous retroviruses and ADHD. World J. Biol. Psychiatry.

[169] Balestrieri E., Cipriani C., Matteucci C., Capodicasa N., Pilika A., Korca I., Sorrentino R., Argaw-Denboba A., Bucci I., Miele M.T. (2016). Transcriptional activity of human endogenous retrovirus in Albanian children with autism spectrum disorders. New Microbiol..

[170] D’Agati E., Pitzianti M., Balestrieri E., Matteucci C., SinibaldiVallebona P., Pasini A. (2016). First evidence of HERV-H transcriptional activity reduction after methylphenidate treatment in a young boy with ADHD. New Microbiol..

[171] Cipriani C., Pitzianti M.B., Matteucci C., D’Agati E., Miele M.T., Rapaccini V., Grelli S., Curatolo P., Sinibaldi-Vallebona P., Pasini A. (2018). The Decrease in Human Endogenous Retrovirus-H Activity Runs in Parallel with Improvement in ADHD Symptoms in Patients Undergoing Methylphenidate Therapy. Int. J. Mol. Sci..

[172] Huang W.J., Liu Z.C., Wei W., Wang G.H., Wu J.G., Zhu F. (2006). Human endogenous retroviral pol RNA and protein detected and identified in the blood of individuals with schizophrenia. Schizophr. Res..

[173] Yao Y., Schröder J., Nellåker C., Bottmer C., Bachmann S., Yolken R.H., Karlsson H. (2008). Elevated levels of human endogenous Retrovirus-W transcripts in blood cells from patients with first episode schizophrenia. Genes Brain Behav..

[174] Perron H., Hamdani N., Faucard R., Lajnef M., Jamain S., Daban-Huard C., Sarrazin S., LeGuen E., Houenou J., Delavest M. (2012). Molecular characteristics of Human Endogenous Retrovirus Type-W in schizophrenia and bipolar disorder. Transl. Psychiatry.

[175] Huang W., Li S., Hu Y., Yu H., Luo F., Zhang Q., Zhu F. (2011). Implication of the env gene of the human endogenous retrovirus W family in the expression of BDNF and DRD3 and development of recent-onset schizophrenia. Schizophr. Bull..

[176] Frank O., Giehl M., Zheng C., Hehlmann R., Leib-Mösch C., Seifarth W. (2005). Human endogenous retrovirus expression profiles in samples from brains of patients with schizophrenia and bipolar disorders. J. Virol..

[177] Karlsson H., Bachmann S., Schröder J., McArthur J., Torrey E.F., Yolken R.H. (2001). Retroviral RNA identified in the cerebrospinal fluids and brains of individuals with schizophrenia. Proc. Natl. Acad. Sci. USA.

[178] Weis S., Llenos I.C., Sabunciyan S., Dulay J.R., Isler L., Yolken R., Perron H. (2007). Reduced expression of human endogenous retrovirus (HERV)-W GAG protein in the cingulate gyrus and hippocampus in schizophrenia, bipolar disorder, and depression. J. Neural Transm..

[179] Perron H., Mekaoui L., Bernard C., Veas F., Stefas I., Leboyer M. (2008). Endogenous retrovirus type W GAG and envelope protein antigenemia in serum of schizophrenic patients. Biol. Psychiatry.

[180] Melbourne J.K., Chase K.A., Feiner B., Rosen C., Sharma R.P. (2018). Long non-coding and endogenous retroviral RNA levels are associated with proinflammatory cytokine mRNA expression in peripheral blood cells: Implications for schizophrenia. Psychiatry Res..

[181] Ibi D., González-Maeso J. (2015). Epigeneticsignaling in schizophrenia. Cell. Signal..

[182] Mak M., Samochowiec J., Frydecka D., Pełka-Wysiecka J., Szmida E., Karpiński P., Sąsiadek M.M., Piotrowski P., Samochowiec A., Misiak B. (2019). First-episode schizophrenia is associated with a reduction of HERV-K methylation in peripheral blood. Psychiatry Res..

[183] Perron H., Lang A. (2010). The human endogenous retrovirus link between genes and environment in multiple sclerosis and in multifactorial diseases associating neuroinflammation. Clin. Rev. Allergy Immunol..

[184] Robbez-Masson L., Rowe H.M. (2015). Retrotransposons shape species-specific embryonic stem cell gene expression. Retrovirology.

[185] Magiorkinis G., Katzourakis A., Lagiou P. (2017). Roles of Endogenous Retroviruses in Early Life Events. Trends Microbiol..

[186] Gropman A.L., Batshaw M.L. (2010). Epigenetics, copy number variation, and other molecular mechanisms underlying neurodevelopmental disabilities: New insights and diagnostic approaches. J. Dev. Behav. Pediatr..

[187] LaSalle J.M., Powell W.T., Yasui D.H. (2013). Epigenetic layers and players underlying neurodevelopment. Trends Neurosci..

[188] Grafodatskaya D., Chung B., Szatmari P., Weksberg R. (2010). Autism spectrum disorders and epigenetics. J. Am. Acad. Child Adolesc. Psychiatry.

[189] Kelley D., Rinn J. (2012). Transposable elements reveal a stem cell-specific class of long noncoding RNAs. Genome Biol..

[190] Cogill S.B., Srivastava A.K., Yang M.Q., Wang L. (2018). Co-expression of long non-coding RNAs and autism risk genes in the developing human brain. BMC Syst. Biol..

[191] Hakim S.T., Alsayari M., McLean D.C., Saleem S., Addanki K.C., Aggarwal M., Mahalingam K., Bagasra O. (2008). A large number of the human microRNAs target lentiviruses, retroviruses, and endogenous retroviruses. Biochem. Biophys. Res. Commun..

[192] Hadjiargyrou M., Delihas N. (2013). The intertwining of transposable elements and non-coding RNAs. Int. J. Mol. Sci..

[193] Wang Y., Xu Z., Jiang J., Xu C., Kang J., Xiao L., Wu M., Xiong J., Guo X., Liu H. (2013). Endogenous miRNA sponge lincRNA-RoR regulates Oct4, Nanog, and Sox2 in human embryonic stem cell self-renewal. Dev. Cell.

[194] Durruthy-Durruthy J., Sebastiano V., Wossidlo M., Cepeda D., Cui J., Grow E.J., Davila J., Mall M., Wong W.H., Wysocka J. (2016). The primate-specific noncoding RNA HPAT5 regulates pluripotency during human preimplantation development and nuclear reprogramming. Nat. Genet..

[195] Lavillette D., Marin M., Ruggieri A., Mallet F., Cosset F.L., Kabat D. (2002). The envelope glycoprotein of human endogenous retrovirus type W uses a divergent family of amino acid transporters/cell surface receptors. J. Virol..

[196] Weis S., Llenos I.C., Dulay J.R., Verma N., Sabunciyan S., Yolken R.H. (2007). Changes in region- and cell type-specific expression patterns of neutral amino acid transporter 1 (ASCT-1) in the anterior cingulate cortex and hippocampus in schizophrenia, bipolar disorder and major depression. J. Neural Transm.

[197] Rojas D.C. (2014). The role of glutamate and its receptors in autism and the use of glutamate receptor antagonists in treatment. J. Neural Transm.

[198] Li S., Liu Z.C., Yin S.J., Chen Y.T., Yu H.L., Zeng J., Zhang Q., Zhu F. (2013). Human endogenous retrovirus W family envelope gene activates the small conductance Ca2+-activated K+ channel in human neuroblastoma cells through CREB. Neuroscience.

[199] Angelucci F., Brenè S., Mathé A.A. (2005). BDNF in schizophrenia, depression and corresponding animal models. Mol. Psychiatry.

[200] Kasarpalkar N.J., Kothari S.T., Dave U.P. (2014). Brain-Derived Neurotrophic Factor in children with Autism Spectrum Disorder. Ann. Neurosci..

[201] Grandi N., Tramontano E. (2018). Human Endogenous Retroviruses Are Ancient Acquired Elements Still Shaping Innate Immune Responses. Front. Immunol..

[202] Hurst T.P., Magiorkinis G. (2015). Activation of the innate immune response by endogenous retroviruses. J. Gen. Virol..

[203] Hurst T.P., Magiorkinis G. (2017). Epigenetic Control of Human Endogenous Retrovirus Expression: Focus on Regulation of Long-Terminal Repeats (LTRs). Viruses.

[204] Aswad A., Katzourakis A. (2012). Paleovirology and virally derived immunity. Trends Ecol. Evol..

[205] Stocking C., Kozak C.A. (2008). Murine endogenous retroviruses. Cell. Mol. Life Sci..

[206] Maksakova I.A., Romanish M.T., Gagnier L., Dunn C.A., van de Lagemaat L.N., Mager D.L. (2006). Retroviral elements and their hosts: Insertional mutagenesis in the mouse germ line. PLoS Genet..

[207] Ribet D., Harper F., Dupressoir A., Dewannieux M., Pierron G., Heidmann T. (2008). An infectious progenitor for the murine IAP retrotransposon: Emergence of an intracellular genetic parasite from an ancient retrovirus. Genome Res..

[208] Magiorkinis G., Gifford R.J., Katzourakis A., De Ranter J., Belshaw R. (2012). Env-less endogenous retroviruses are genomic superspreaders. Proc. Natl. Acad. Sci. USA.

[209] Rowe H.M., Trono D. (2011). Dynamic control of endogenous retroviruses during development. Virology.

[210] Kuff E.L., Fewell J.W. (1985). Intracisternal A-particle gene expression in normal mouse thymus tissue: Gene products and strain-related variability. Mol. Cell. Biol..

[211] Wang X.Y., Steelman L.S., McCubrey J.A. (1997). Abnormal activation of cytokine gene expression by intracisternal type A particle transposition: Effects of mutations that result in autocrine growth stimulation and malignant transformation. Cytokines Cell. Mol. Ther..

[212] Howard G., Eiges R., Gaudet F., Jaenisch R., Eden A. (2008). Activation and transposition of endogenous retroviral elements in hypomethylation induced tumors in mice. Oncogene.

[213] Sharif J., Shinkai Y., Koseki H. (2013). Is there a role for endogenous retroviruses to mediate long-term adaptive phenotypic response upon environmental inputs?. Philos. Trans. R. Soc. Lond B Biol. Sci..

[214] Ribet D., Dewannieux M., Heidmann T. (2004). An active murine transposon family pair: Retrotransposition of “master” MusD copies and ETn trans-mobilization. Genome Res..

[215] Ribet D., Harper F., Dewannieux M., Pierron G., Heidmann T. (2007). Murine MusD retrotransposon: Structure and molecular evolution of an “intracellularized” retrovirus. J. Virol..

[216] Maksakova I.A., Mager D.L. (2005). Transcriptional regulation of early transposon elements, an active family of mouse long terminal repeat retrotransposons. J. Virol..

[217] Dupressoir A., Marceau G., Vernochet C., Bénit L., Kanellopoulos C., Sapin V., Heidmann T. (2005). Syncytin-A and syncytin-B, two fusogenic placenta-specific murine envelope genes of retroviral origin conserved in Muridae. Proc. Natl. Acad. Sci. USA.

[218] Dupressoir A., Vernochet C., Harper F., Guégan J., Dessen P., Pierron G., Heidmann T. (2011). A pair of co-opted retroviral envelope syncytin genes is required for formation of the two-layered murine placental syncytiotrophoblast. Proc. Natl. Acad. Sci. USA.

[219] Redelsperger F., Raddi N., Bacquin A., Vernochet C., Mariot V., Gache V., Blanchard-Gutton N., Charrin S., Tiret L., Dumonceaux J. (2016). Genetic Evidence That Captured Retroviral Envelope syncytins Contribute to Myoblast Fusion and Muscle Sexual Dimorphism in Mice. PLoS Genet..

[220] Søe K., Hobolt-Pedersen A.S., Delaisse J.M. (2015). The elementary fusion modalities of osteoclasts. Bone.

[221] Ergaz Z., Weinstein-Fudim L., Ornoy A. (2016). Genetic and non-genetic animal models for autism spectrum disorders (ASD). Reprod. Toxicol..

[222] Scattoni M.L., Ricceri L., Crawley J.N. (2011). Unusual repertoire of vocalizations in adult BTBR T+tf/J mice during three types of social encounters. Genes Brain Behav..

[223] Hava G., Vered L., Yael M., Mordechai H., Mahoud H. (2006). Alterations in behaviour in adult offspring mice following maternal inflammation during pregnancy. Dev. Psychobiol..

[224] Malkova N.V., Yu C.Z., Hsiao E.Y., Moore M.J., Patterson P.H. (2012). Maternal immune activation yields offspring displaying mouse versions of the three core symptoms of autism. Brain Behav. Immun..

[225] Smith S.E., Li J., Garbett K., Mirnics K., Patterson P.H. (2007). Maternal immune activation alters fetal brain development through interleukin-6. J. Neurosci..

[226] Weber-Stadlbauer U., Richetto J., Labouesse M.A., Bohacek J., Mansuy I.M., Meyer U. (2017). Transgenerational transmission and modification of pathological traits induced by prenatal immune activation. Mol. Psychiatry.

[227] Choi G.B., Yim Y.S., Wong H., Kim S., Kim H., Kim S.V., Hoeffer C.A., Littman D.R., Huh J.R. (2016). The maternal interleukin-17a pathway in mice promotes autism-like phenotypes in offspring. Science.

[228] Deckmann I., Schwingel G.B., Fontes-Dutra M., Bambini-Junior V., Gottfried C. (2018). Neuroimmune Alterations in Autism: A Translational Analysis Focusing on the Animal Model of Autism Induced by Prenatal Exposure to Valproic Acid. Neuroimmunomodulation.

[229] Wagner G.C., Reuhl K.R., Cheh M., McRae P., Halladay A.K. (2006). A new neurobehavioral model of autism in mice: Pre- and postnatal exposure to sodium valproate. J. Autism Dev. Disord..

[230] Nicolini C., Fahnestock M. (2018). The valproic acid-induced rodent model of autism. Exp. Neurol..

[231] Cipriani C., Ricceri L., Matteucci C., De Felice A., Tartaglione A.M., Argaw-Denboba A., Pica F., Grelli S., Calamandrei G., Sinibaldi Vallebona P. (2018). High expression of Endogenous Retroviruses from intrauterine life to adulthood in two mouse models of Autism Spectrum Disorders. Sci. Rep..

[232] Tartaglione A.M., Cipriani C., Chiarotti F., Perrone B., Balestrieri E., Matteucci C., Sinibaldi-Vallebona P., Calamandrei G., Ricceri L. (2019). Early Behavioral Alterations and Increased Expression of Endogenous Retroviruses Are Inherited Across Generations in Mice Prenatally Exposed to Valproic Acid. Mol. Neurobiol..

[233] Konopko M.A., Densmore A.L., Krueger B.K. (2017). Sexually Dimorphic Epigenetic Regulation of Brain-Derived Neurotrophic Factor in Fetal Brain in the Valproic Acid Model of Autism Spectrum Disorder. Dev. Neurosci..

[234] Tung E.W., Winn L.M. (2010). Epigenetic modifications in valproic acid-induced teratogenesis. Toxicol. Appl. Pharmacol..

[235] Holliday R. (1998). The possibility of epigenetic transmission of defects induced by teratogens. Mutat. Res..

[236] Gagnier L., Belancio V.P., Mager D.L. (2019). Mouse germ line mutations due to retrotransposon insertions. Mob. DNA.

[237] Guliyev M., Yilmaz S., Sahin K., Marakli S., Gozukirmizi N. (2013). Human endogenous Retrovirus-H insertion screening. Mol. Med. Rep..

[238] CakmakGuner B., Karlik E., Marakli S., Gozukirmizi N. (2018). Detection of HERV-K6 and HERV-K11 transpositions in the human genome. Biomed. Rep..

[239] Garcia-Montojo M., Dominguez-Mozo M., Arias-Leal A., Garcia-Martinez Á., De lasHeras V., Casanova I., Faucard R., Gehin N., Madeira A., Arroyo R. (2013). The DNA copy number of human endogenous Retrovirus-W (MSRV-type) is increased in multiple sclerosis patients and is influenced by gender and disease severity. PLoS ONE.

[240] Bundo M., Toyoshima M., Okada Y., Akamatsu W., Ueda J., Nemoto-Miyauchi T., Sunaga F., Toritsuka M., Ikawa D., Kakita A. (2014). Increased l1 retrotransposition in the neuronal genome in schizophrenia. Neuron.

[241] Nomura J., Takumi T. (2012). Animal models of psychiatric disorders that reflect human copy number variation. Neural Plast..

[242] Nishioka M., Bundo M., Iwamoto K., Kato T. (2019). Somatic mutations in the human brain: Implications for psychiatric research. Mol. Psychiatry.

